# *Neisseria gonorrhoeae* infects the heterogeneous epithelia of the human cervix using distinct mechanisms

**DOI:** 10.1371/journal.ppat.1008136

**Published:** 2019-12-02

**Authors:** Qian Yu, Liang-Chun Wang, Sofia Di Benigno, Scott D. Gray-Owen, Daniel C. Stein, Wenxia Song

**Affiliations:** 1 Department of Cell Biology & Molecular Genetics, University of Maryland, College Park, Maryland, United States of America; 2 Department of Marine Biotechnology and Resources, National Sun Yat-sen University, Kaohsiung, Taiwan; 3 Department of Molecular Genetics, University of Toronto, Toronto, Ontario, Canada; University of Oxford, UNITED KINGDOM

## Abstract

Sexually transmitted infections are a critical public health issue. However, the mechanisms underlying sexually transmitted infections in women and the link between the infection mechanism and the wide range of clinical outcomes remain elusive due to a lack of research models mimicking human infection *in vivo*. We established a human cervical tissue explant model to mimic local *Neisseria gonorrhoeae* (GC) infections. We found that GC preferentially colonize the ectocervix by activating integrin-β1, which inhibits epithelial shedding. GC selectively penetrate into the squamocolumnar junction (TZ) and endocervical epithelia by inducing β-catenin phosphorylation, which leads to E-cadherin junction disassembly. Epithelial cells in various cervical regions differentially express carcinoembryonic antigen-related cell adhesion molecules (CEACAMs), the host receptor for GC opacity-associated proteins (Opa_CEA_). Relatively high levels were detected on the luminal membrane of ecto/endocervical epithelial cells but very low levels intracellularly in TZ epithelial cells. CEACAM-Opa_CEA_ interaction increased ecto/endocervical colonization and reduced endocervical penetration by increasing integrin-β1 activation and inhibiting β-catenin phosphorylation respectively, through CEACAM downstream signaling. Thus, the intrinsic properties of cervical epithelial cells and phase-variation of bacterial surface molecules both play a role in controlling GC infection mechanisms and infectivity, preferential colonization or penetration, potentially leading to asymptomatic or symptomatic infection.

## Author summary

Gonorrhea, caused by *Neisseria gonorrhoeae* (GC), is a common sexually transmitted infection and has become a public health crisis due to a steady increase in antibiotic-resistant cases. How GC infect the female reproductive tract (FRT) and cause various clinical outcomes is still unknown. This study used human cervical explants to examine the mechanism by which GC infect the heterogeneous mucosa of the human cervix, the gate of the FRT. We show that GC preferentially colonize stratified epithelial cells in the vaginal-cervical region by enhancing epithelial cell adherence. GC selectively penetrate into tissues of the squamocolumnar junction and cervical-endometrial regions but not the vaginal-cervical region by loosening epithelial cell-cell junctions. The expression of opacity-associated proteins on GC regulates the infection patterns depending on the expression of the host receptors on various types of epithelia and receptor downstream signaling. These data suggest that both intrinsic properties of cervical epithelial cells and phase-variation of bacterial surface molecules play a role in determining GC infectivity. Our findings can explain how GC infection leads to asymptomatic or symptomatic infection.

## Introduction

Sexually transmitted infections (STIs) remain a challenging public health issue due, in part, to a lack of vaccines for many STI pathogens. Gonorrhea, caused by the Gram-negative bacterium *Neisseria gonorrhoeae* (GC), is a common STI [1] and has reemerged as a public health crisis due to an upsurge of multidrug-resistant strains [2, 3]. Most infected women are asymptomatic [4, 5], leading to treatment delay, silent transmission, and susceptibility to complications including pelvic inflammatory disease (PID), infertility, and a predisposition to life-threatening ectopic pregnancy. However, how STI pathogens interact with the human female reproductive tract (FRT) and cause a wide range of clinical outcomes remains elusive. A primary obstacle for a better understanding of STIs is the lack of infection models that mimic human infection.

GC infect humans exclusively and initiate infection in the FRT at the cervix, the gateway of the FRT [6]. Clinical studies suggest that GC colonization at the mucosal surface of the vagina and ectocervix leads to asymptomatic local infection [5]. Subepithelial GC have only been found in biopsies from the endocervix and the squamocolumnar junction (transformational zone, TZ) of symptomatic patients [7]. The mechanism by which GC interactions with the cervical mucosal surface cause either symptomatic or asymptomatic infection remains unknown.

The mucosal surface of the human cervix varies significantly and is generally divided into three regions: the ectocervix, consisting of multilayered, non-polarized, stratified, squamous epithelial cells; the endocervix, containing a single-layer of polarized, columnar cells; and the TZ, where epithelial cells progressively change from stratified squamous to columnar cells [8]. Cells in these regions have different properties. In addition to cell morphology and polarity, one of the distinguishing features between squamous and columnar epithelial cells are their adherens and apical cell-cell junctions, respectively. The adherens junction is formed by E-cadherin homo-interactions between neighboring cells, and the apical junction consists of both adherens and tight junction complexes [9]. While both types of junctions are critical for the epithelial barrier function, each has unique functional and regulatory properties [9]. How the epithelial heterogeneity of the cervix impacts GC infection and clinical outcomes has not been examined.

Bacteria have evolved multiple mechanisms to overcome mucosal surfaces. One mechanism is phase variation. GC can turn on and off pilin expression [10] and express any of 11 opacity-associated (Opa) protein isoforms at any time [11]. Early studies show that pili initiate GC contact with epithelial cells [12] and Opa mediates intimate GC-epithelial interactions by binding to host receptors [13–15]. Studies, based on human primary cells, biopsies and fallopian tube organ culture, identified human-specific receptors for GC surface molecules. Pili bind to complement receptor 3 (CR3), a β_2_ integrin expressed on primary cervical epithelial cells, which is essential for GC adherence [16, 17]. Most Opa isoforms bind to carcinoembryonic antigen-related cell adhesion molecules (CEACAMs) (Opa_CEA_) while one binds to heparan sulfate proteoglycans (HSPG) (Opa_HSPG_) [18–21]. However, the human upper and lower FRT expresses different CEACAM isoforms [22]. CEACAMs can induce cell-cell adhesion or signal through oligomerization [23]. The cytoplasmic tail of CEACAM1, the only transmembrane CEACAM that is expressed in human cervical epithelial cells, contains an immune-receptor tyrosine-based inhibitory motif (ITIM) [24]. The ITIM can inhibit signaling by activating the SH2 domain-containing nonreceptor tyrosine phosphatases SHP1/2 [25, 26]. The role of this ITIM and its downstream SHP in GC infection is unclear.

Defining the role of interactions between GC surface structures and their human-specific receptors in GC pathogenesis has been problematic, as the data suggest different roles depending on the infection model and the phenotype of GC used. GC Opa_CEA_ but not Opa_HSPG_ induces integrin activation in non-polarized human epithelial cell lines and ectocervical epithelial cells of CEACAM-expressing transgenic mice, inhibiting their shedding and increasing GC colonization [27, 28]. While Opa_CEA_ expression has been reported to promote transcellular transcytosis of GC across polarized colonic epithelial cells [29], we have shown that Opa_CEA_ expression inhibits GC penetration into the human endocervical epithelium [30]. As both the surfaces of the human cervix and GC are heterogeneous, different GC variants could trigger different host responses at the three cervical regions, due to changes in the host receptors engaged by GC, leading to different infectivity and clinical outcomes.

To examine the mechanism by which GC interact with the heterogeneous mucosal surfaces, we established human cervical tissue explants, which preserve the distinct properties of each cervical region *in vivo* and mimic GC infection as observed in patient biopsies. This infection model enabled us to show that the outcome of GC infection depends on the type of cervical epithelial cells the bacteria interact with and the variant of Opa GC express. GC vary infectivity by differentially regulating integrin β1, β-catenin, and E-cadherin in various epithelial cells of the three cervical regions. Our results potentially provide a mechanistic explanation for the wide range of clinical outcomes following GC infection in women.

## Results

### GC exhibit distinct infectivity patterns on different mucosal surfaces of the human cervix

To examine GC infection in the human cervix, we cultured human cervical tissues surgically removed from 28–40 year-old women as an *ex vivo* model for GC infection, using a modified version of a published protocol [31]. We compared the morphology and organization of cervical epithelia in explants before and after cultured for 3 days, a preparation period for infection, using immunofluorescence and three-dimensional confocal fluorescence microscopy (3D-CFM). Cryopreserved tissues were sectioned across both the luminal and basal surfaces of the epithelium. Epithelial cells were identified by cytokeratin staining and their cell-cell junctions by E-cadherin staining (S1 Fig). The three regions of the cervix were identified by the distinct properties of epithelial cells: 1) stratified, multilayered, and non-polarized epithelial cells in the ectocervix, 2) multilayered and non-polarized cells in the TZ, and 3) columnar, single-layered, and polarized cells in the endocervix. These properties were unchanged after the 3-day culture period (S1 Fig and S1 Video).

**Fig 1:**
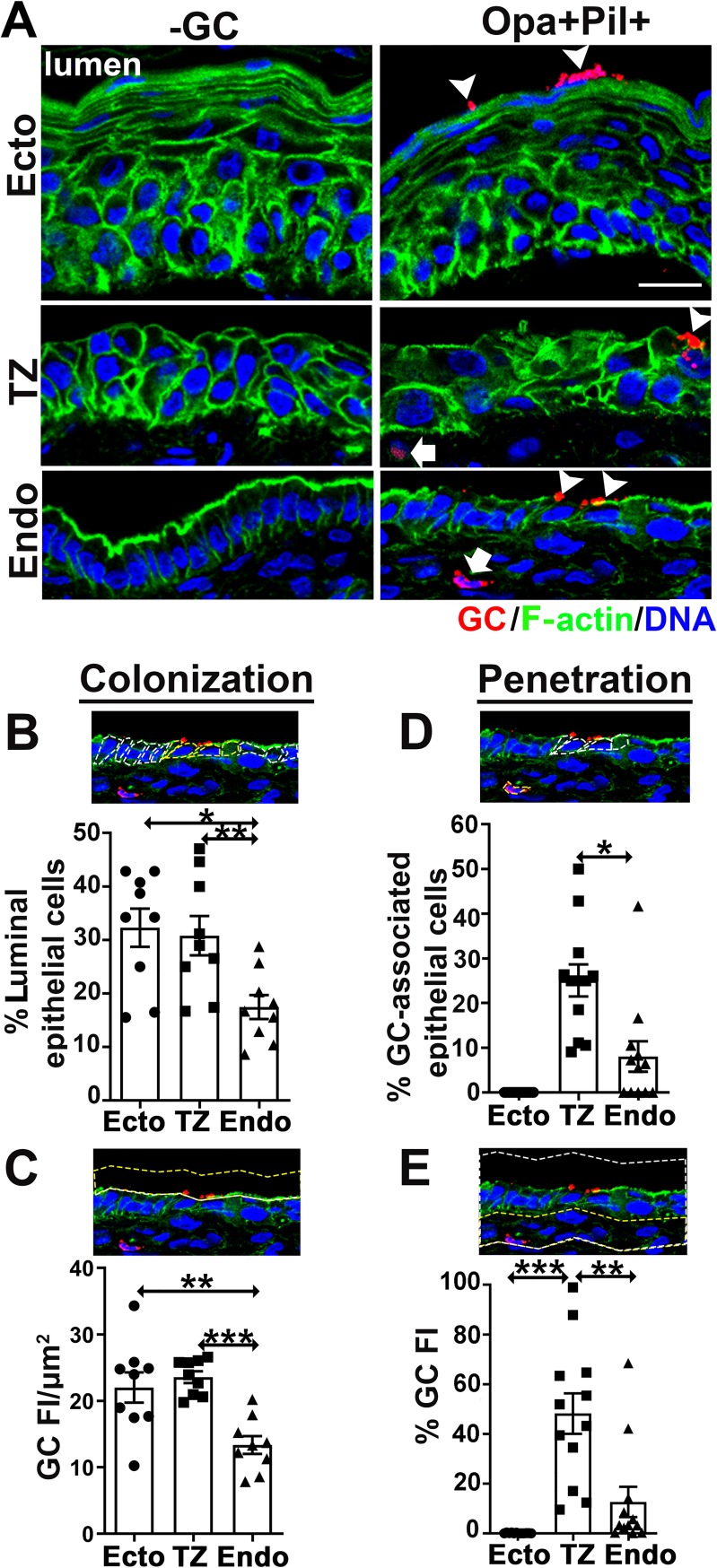
GC exhibit different levels of colonization and subepithelial penetration in the ectocervical, TZ, and endocervical epithelia. Human cervical tissue explants were incubated with MS11 Pil+Opa+ for 24 h (MOI~10), washed at 6 and 12 h post-inoculation, and cryopreserved. Tissue sections were collected, processed, and analyzed using CFM. (A) Representative images of the three regions of cervical tissue explants with or without GC infection. Scale bar, 20 μm. Arrowheads, colonizing GC. Arrows, penetrating GC. See also S2 Video. (B and C) Quantification of GC colonization by the percentage (±SEM) of luminal epithelial cells with GC attaching to the luminal surface (B, white dashed lines circling individual luminal epithelial cells and yellow dashed lines circling GC-associated luminal epithelial cells) and by fluorescence intensity (FI) (±SEM) of GC staining per μm^2^ of the luminal surface (C, yellow dashed lines circling the luminal surface area where GC FI was measured). Shown are the averages from 3 independent analyses of cervical tissues from 3 human subjects. (D and E) Quantification of GC penetration by the percentage (±SEM) of GC-associated epithelial cells with GC at subepithelia (D, white dashed lines circling epithelial cells with GC colonization and yellow dashed lines circling cells with subepithelial GC) and by the percentage (±SEM) of GC FI at the subepithelium relative to the total GC FI at the epithelium (E, white dashed lines circling both epithelial and subepithelial regions and yellow dashed lines circling the subepithelial region). Shown are the means from 12 images per cervical region acquired from 2~3 independent analyses of 3 human cervixes. *p<0.05; **p<0.01; ***p<0.001, as determined by Student’s t-test and one-way non-parametric ANOVA (Kruskal-Wallis test).

**Fig 2:**
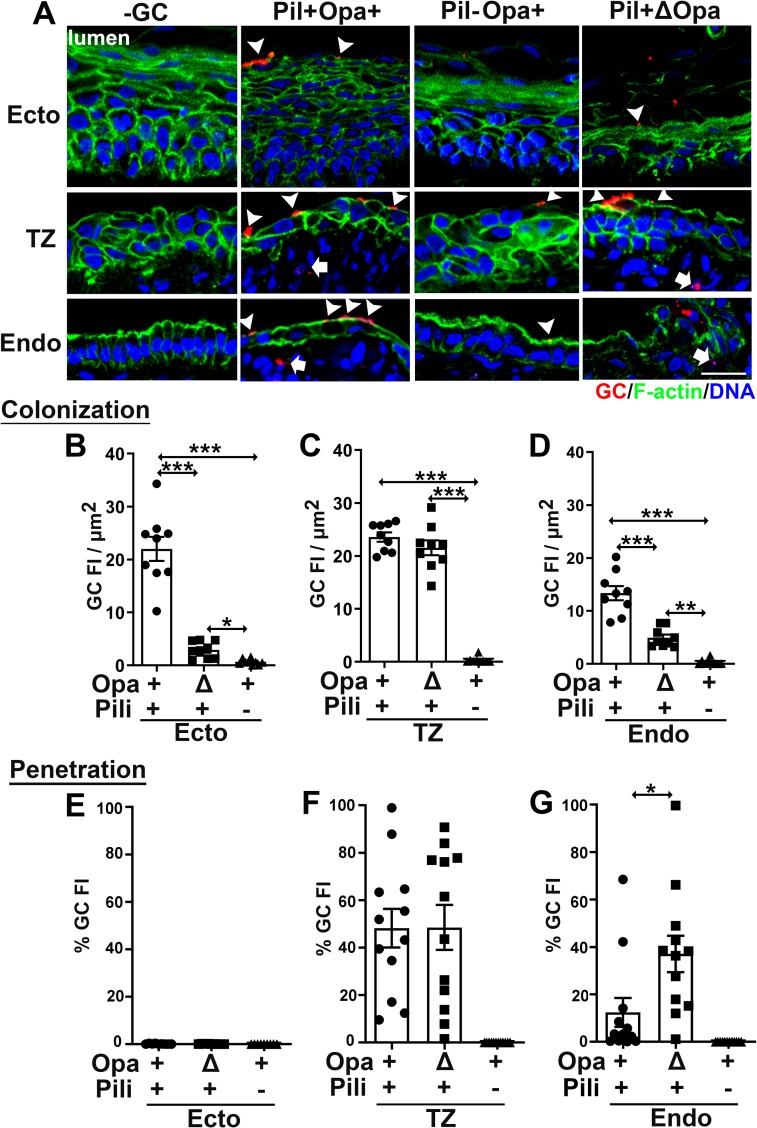
Pili are required for GC colonization, and Opa proteins play distinct roles in GC infection in different regions of the human cervix. Human cervical tissue pieces were inoculated with Pil+Opa+, Pil+ΔOpa, or Pil-Opa+ GC for 24 h (MOI~10), washed at 6 and 12 h post-inoculation and cryopreserved. Tissue sections were collected, processed, and analyzed using CFM. (A) Representative images. Arrowheads, colonizing GC. Arrows, penetrating GC. Scale bar, 20 μm. (B-D) Levels of GC colonization at the ectocervix (B), TZ (C), and endocervix (D) were quantified by GC FI per μm^2^ (±SEM). Shown are the averages from 3 independent analyses of cervical tissues from 3 human subjects. (E-G) Levels of GC penetration into the subepithelia of the ectocervix (E), TZ (F), and endocervix (G) by the percentage (±SEM) of GC FI at the subepithelia relative to the total GC FI at the epithelium. Shown are the means from 12 images per cervical region acquired from 2~3 independent analyses of each of 3 human cervixes. *p<0.05; **p< 0.01; ***p<0.001, as determined by Student’s t-test and one-way non-parametric ANOVA (Kruskal-Wallis test).

To determine the infection pattern in the various cervical regions, we incubated tissue slices from the same human subject with or without MS11 GC that were piliated and expressed phase variable Opa (Pil+Opa+) for 24 h, with an initial MOI of ~10 bacteria/luminal epithelial cell. Each tissue slice contained all the three regions of the cervix. Inoculated tissue explants were rinsed at 6 and 12 h to remove unassociated GC. Tissue sections that crossed both the luminal and basal surfaces of epithelia were stained for GC, F-actin, and nuclei, and imaged by CFM (S2 Fig). We designed two methods to quantify GC colonization using CFM images (Fig 1A, arrowheads): 1) the percentage of luminal epithelial cells with GC staining (Fig 1B) and 2) the fluorescence intensity (FI) of GC staining per μm^2^ of the luminal surface (Fig 1C). The two methods consistently showed that GC colonized all three regions of the human cervix, with the colonization levels at the ectocervix and TZ significantly higher than that at the endocervix (Fig 1B and 1C). We designed similar methods to quantify GC penetration into the subepithelia (Fig 1A, arrows): 1) the percentage of GC-associated cells with GC staining at the subepithelium (Fig 1D) and 2) the percentage of GC FI in the subepithelium (Fig 1E). Subepithelial GC were only detected in the TZ and the endocervix but not in the ectocervix (Fig 1A, 1D and 1E and S2 Video), with a higher level of GC at the subepithelium of the TZ than the endocervix (Fig 1D and 1E). Thus, GC do not interact with the various epithelia equally. When the mucosal surface of all three cervical regions is available, GC preferentially colonize the ectocervix and TZ and selectively penetrate into the TZ and the endocervix.

### Opa proteins but not pili have distinct roles in GC infection in different regions of the human cervix

To examine the roles of Opa and pili in cervical infection, we compared the infectivity of piliated (Pil+) and nonpiliated (Pil-) GC with phase variable Opa (wild type, Opa+) or all 11 Opa isoforms genetically deleted (ΔOpa), using the methods described above. We found that Pil-Opa+ GC failed to colonize efficiently any of the three cervical mucosal regions (Fig 2A–2D). Deletion of Opa only reduced GC colonization at the ecto/endocervix but not at the TZ (Fig 2A–2D). No Pil-Opa+ GC were detected in the subepithelia of the cervix (Fig 2A and 2E–2G), and no GC, no matter if they expressed pili or Opas, was found in the subepithelium of the ectocervix (Fig 2A and 2E). Deletion of Opa increased the amount of GC penetrating into the endocervical but not the ectocervical and the TZ epithelia (Fig 2A and 2E–2G). These results suggest that pili are essential for GC colonization of all types of cervical epithelial cells, while Opa proteins promote GC adherence to the ecto/endocervix but impede penetration into endocervical tissues.

**Fig 3:**
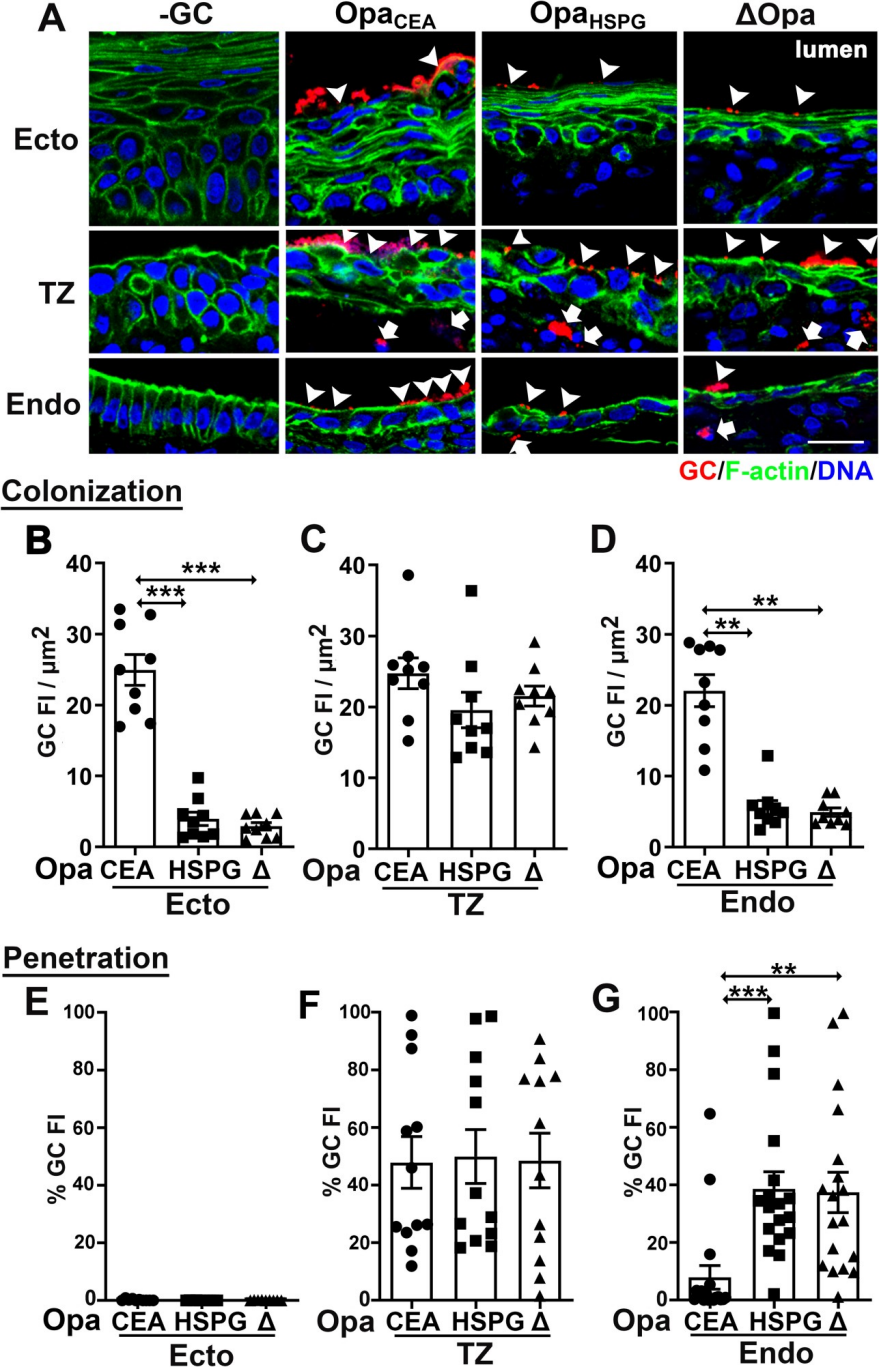
Opa variants differentially regulate GC infectivity in the three regions of the human cervix. Human cervical tissue pieces were incubated with Pil+ Opa_CEA_, Opa_HSPG_, and ΔOpa GC for 24 h (MOI~10), washed at 6 and 12 h post-inoculation and cryopreserved. Tissue sections were collected, processed, and analyzed using CFM. (A) Representative images. Arrowheads, colonizing GC. Arrows, penetrating GC. Scale bar, 20 μm. (B-D) Levels of GC colonization at the ectocervix (B), TZ (C), and endocervix (D) were quantified by GC FI per μm^2^. Shown were the means from randomly selected images from 3 independent analyses of each of 3 human cervixes. (E-G) Levels of GC penetration into the subepithelia of the ectocervix (E), TZ (F), and endocervix (G) by the percentage (±SEM) of GC FI at the subepithelia relative to the total GC FI at the epithelium. Shown are the means from 12~18 images per cervical region acquired from 3 independent analyses of each of 3 human cervixes. **p< 0.01; ***p<0.001, as determined by Student’s t-test and one-way non-parametric ANOVA (Kruskal-Wallis test).

A majority of Opas bind to CEACAMs (Opa_CEA_) while one binds to HSPGs (Opa_HSPG_) [32]. To determine if the two groups of Opas differentially regulate colonization and penetration, we utilized isogenic strains of Pil+ GC that express a well-defined Opa_CEA_ (OpaH) or Opa_HSPG_ (OpaC) that cannot phase vary [33, 34]. Compared to the ΔOpa strain, the expression of Opa_CEA_ significantly increased the level of GC colonization at the ecto/endocervix while Opa_HSPG_ did not (Fig 3A, 3B and 3D). In contrast, the expression of Opa_CEA_ but not Opa_HSPG_ exclusively reduced GC penetration into the endocervix (Fig 3G). Opa_HSPG_ GC colonized and penetrated at the same levels as ΔOpa GC in all regions of the cervix (Fig 3B–3G). Interestingly, the levels of both GC colonization and penetration at the TZ remained the same independently of whether GC expressed Opa_CEA_, Opa_HSPG_, or no Opa (Fig 3C and 3F), supporting our above finding that the pilus alone determines GC colonization and penetration in the TZ (Fig 2C and 2F). Expression of either a well-defined Opa_CEA_ (OpaH) or Opa_HSPG_ (OpaC) (32, 52) also did not promote GC penetration into the ectocervical subepithelium (Fig 3E), confirming that the absence of penetration seen with the parental wt strain Pil+Opa+ (Fig 2E) was not due to it lacking expression of an Opa_CEA_ or an Opa_HSPG_. These results indicate that Opa_CEA_ selectively enhances GC colonization at the ecto/endocervix and suppresses GC penetration into the endocervical subepithelium, while Opa_HSPG_ is not significantly involved in GC colonization of the human cervix. Thus, phase variable-expression of Opa isoforms differentially impact infectivity at the three cervical regions.

### Heterogeneous expression levels of CEACAMs on cervical epithelial cells regulate GC infectivity

**Fig 4:**
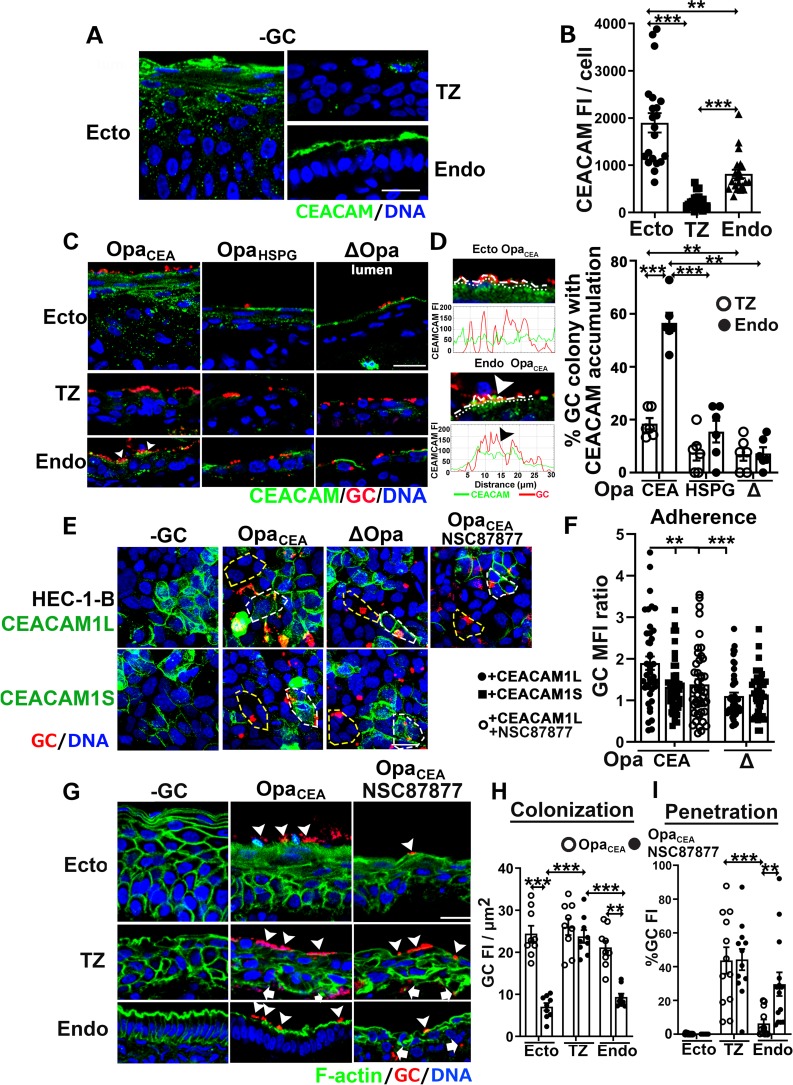
Heterogeneous expression levels of CEACAMs on cervical epithelial cells regulate GC infectivity. (A) Shown are representative CFM images of uninfected human cervical tissue explants stained with anti-CEACAM antibody (reacting with CEACAM 1, 3, and 6). See also S3 Video. (B) The levels of CEACAM expression were measured by the mean (±SEM) of CEACAM FI per cell, generated from 21 images per cervical region acquired from 2~4 independent analyses of 3 human cervixes. (C-D) Tissue explants were inoculated with piliated Opa_CEA_, Opa_HSPG_, and ΔOpa GC for 24 h (MOI~10), washed at 6 and 12 h post-inoculation and cryopreserved. Tissue sections were collected, processed, stained for GC and CEACAMs, and analyzed using CFM. Shown are representative CFM images (C). Arrowhead, GC microcolonies colocalize with CEACAM staining accumulated underneath. The spatial relationship of GC with CEACAMs was quantified by the percentage of GC microcolonies with CEACAM staining accumulation underneath or nearby using FI line profiles of GC and CEACAMs (D). Shown are the means from 2 independent analyses of cervical tissues from 3 human subjects. (E-F) HEC-1-B cells were transiently transfected with or without CEACAM1L or 1S and infected with Pil+Opa_CEA_ or Pil+ΔOpa GC (MOI~10) for 6 h in the absence or presence of the SHP1/2 inhibitor, NSC878777 (20 μM). Shown are representative CFM images (E). White dashed lines circle CEACAM-expressing cells. Yellow dashed lines circle cells that do not express CEACAMs. The levels of GC adherence to CEACAM1L or 1S-expressing cells were compared to those that did not express CEACAMs by the MFI ratio (±SEM) of GC on CEACAM-positive cells versus CEACAM-negative cells in the same images (F). Shown were the mean of 45 CEACAM1-positive regions and the corresponding CEACAM1-negative regions in 15 images acquired in 3 independent experiments. (G-I) Cervical tissue explants were inoculated with Pil+Opa_CEA_ GC in the presence or absence of the SHP inhibitor NSC87877 (20 μM) for 24 h, and tissue sections were stained for GC, F-actin, and DNA. Shown are representative CFM images (G). Arrowheads, colonizing GC, and arrows, penetrating GC. The levels of GC colonization at the ectocervix, TZ and endocervix were quantified by GC FI per μm^2^ (±SEM) of the luminal surface (H), and the levels of GC penetration into the subepithelia of the ectocervix, TZ and endocervix were measured as the percentage (±SEM) of GC FI at the subepithelium (I). Shown are the means from cervical tissues of 3 human subjects as described in Fig 1. Scale bar, 20 μm. **p< 0.01; ***p<0.001, as determined by Student’s t-test and one-way non-parametric ANOVA (Kruskal-Wallis test).

The differential effects of Opa_CEA_ on GC infectivity at various regions of the human cervix led us to hypothesize that epithelial cells at different regions of the human cervix do not express the same levels of CEACAMs. To test this, we stained human cervical tissues with a monoclonal antibody that reacts with CEACAM1, 3 and 6 [35]. CFM images showed strong luminal surface staining and punctate cytoplasmic staining of CEACAMs at the ectocervix and strong apical surface staining alone in the endocervix. However, only sparse puncta of CEACAM staining were observed in the cytoplasm of TZ epithelial cells (Fig 4A and S3 Video). We evaluated CEACAM expression levels by measuring the average values of CEACAM FI per epithelial cell. Among the three regions, the ectocervical epithelial cells exhibited the highest level of CEACAM expression. The endocervical and TZ epithelial cells had ~50% and ~11% of the CEACAM FI as the ectocervical cells, respectively (Fig 4B). The very low expression and the cytoplasmic location of CEACAMs in TZ epithelial cells explain the unresponsiveness of GC infectivity in the TZ to Opa_CEA_ expression.

To determine if Opa_CEA_ regulates infectivity through the host CEACAMs, we determined the percentage of GC colonies recruiting CEACAMs to adherent sites using CFM images (Fig 4C) and FI line profiles of GC and CEACAM staining at the luminal surface (Fig 4D, left panels). We only observed an accumulation of CEACAM staining under microcolonies expressing Opa_CEA_ in the endocervical epithelial cells (Fig 4C, bottom left panel, Fig 4D, and S4 Video, right panel). No significant accumulation of CEACAMs was observed in the endocervix infected by GC expressing Opa_HSPG_ or no Opa (Fig 4C, bottom middle and right panels, and Fig 4D), or in the TZ that expressed a low level of CEACAMs (Fig 4C, middle roll panels, Fig 4D, and S4 Video, middle panel). These results suggest possible direct interactions between Opa_CEA_ and CEACAMs. However, GC, including the Opa_CEA_-expressing strain, did not recruit CEACAMs at the ectocervix, even though the lumenal expression of CEACAMs was maintained in the infected ectocervix (Fig 4C, top panels, and S4 Video, left panel).

To determine the impact of CEACAM expression on GC infectivity, we compared the levels of GC adherence to human endometrial epithelial cells, HEC-1-B, that do or do not express CEACAMs [36]. Among CEACAMs expressed in epithelial cells, CEACAM1 is the only one that has a cytoplasmic domain. Its long spliced isoform (CEACAM1L) contains the ITIM in the cytoplasmic domain, while the short isoform (CEACAM1S) does not [37]. We transiently expressed each of the two isoforms in HEC-1-B cells and compared GC adherence using GC MFI ratios between CEACAM1-expressing and nonexpressing cells in the same images and the same size of areas. We found that the expression of CEACAM1L but not CEACAM1S significantly increased the adherence of Pil+Opa_CEA_ GC after 6-h incubation (Fig 4E and 4F). In contrast, the expression of CEACAM1L or 1S had no significant effect on the adherence level of ΔOpa GC (Fig 4E and 4F). These data suggest that the role of Opa_CEA_ in GC infectivity requires the expression of CEACAM1L.

Our finding that the expression of CEACAM1L but not CEACAM1S in epithelial cells enhances Opa_CEA_ GC adherence suggested a role for the ITIM in the cytoplasmic domain of CEACAM1L. As CEACAM1L activates the tyrosine phosphatases SHP1/2 via its ITIM [24, 26, 38], we examined the effect of inhibiting the enzymatic activity of SHP1/2 with a cell-permeable 7-aza-8-hydroxyquinoline compound, NSC-87877, on GC infectivity. Treatment with the SHP inhibitor abolished the enhancing effects of CEACAM1L expression in HEC-1-B cells on GC adherence (Fig 4E and 4F). Furthermore, SHP inhibition also eliminated the enhancing effect of Opa_CEA_ on GC colonization of the ecto/endocervix (Fig 4G and 4H) as well as the inhibitory effect of Opa_CEA_ on GC penetration into the endocervix (Fig 4G and 4I), without changing the GC growth rate (S3 Fig). In contrast, SHP inhibition did not change the colonization and penetration levels of Pil+Opa_CEA_ in the TZ (Fig 4G–4I). Similarly, the inhibitor treatment blocked Opa_CEA_-mediated inhibition of GC transmigration across polarized T84 human colonic epithelial cells, which express endogenous CEACAMs [29] (S4 Fig). These data suggest that Opa_CEA_ enhances GC colonization and inhibits GC penetration into cervical subepithelia probably by inducing CEACAM1L-mediated activation of SHP.

### Heterogeneous properties of cervical epithelial cells regulate the levels of GC-induced epithelial shedding

**Fig 5:**
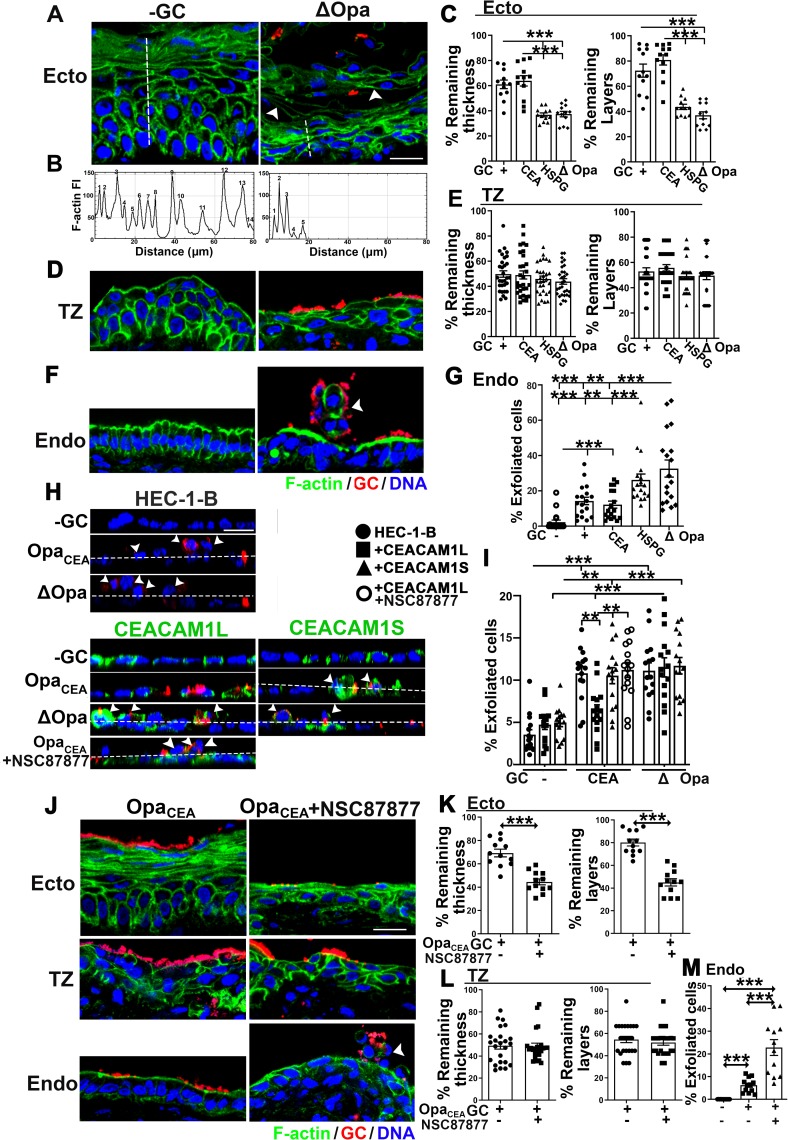
Heterogeneous properties of cervical epithelial cells regulate the levels of GC-induced epithelial shedding. (A-G) Tissue explants were inoculated with or without Pil+Opa+, Opa_CEA_, Opa_HSPG_, or ΔOpa GC for 24 h (MOI~10), washed at 6 and 12 h post-inoculation, and cryopreserved. Tissue sections were collected, processed, and analyzed using CFM. Shown are representative images of the ectocervical (A), TZ (D), and endocervical (F) regions of tissue explants with or without Pil+ΔOpa GC. Arrowheads, shedding cells. (B) Representative line profiles of F-actin FI along dashed lines in A, indicating the number of epithelial layers and the thickness of the epithelium remained. (C and E) The levels of epithelial shedding in the ectocervix (C) and the TZ (E) were quantified by the percentage (±SEM) of the thickness of the epithelium and the number of epithelial layers remained. Shown are the means from 12 (C) or 30 (E) images acquired for each cervical region from 2~3 independent analyses of 3 human cervixes. (G) The levels of epithelial shedding in the endocervix quantified by the percentage (±SEM) of epithelial cells moving above the epithelial monolayer. Shown are the means from 18 images acquired from 2~3 independent analyses of 3 human cervixes. (H-I) HEC-1-B cell monolayers that transfected with or without CEACAM1L or 1S were incubated with or without Pil+Opa_CEA_ or Pil+ΔOpa GC and the SHP1/2 inhibitor for 6 h. Shown are representative images (H). Dashed lines, the surface of monolayers. Arrowheads, cells moving above the epithelial monolayer. Shedding of HEC-1-B cells was quantified by the percentage (±SEM) of cells moving above the monolayers from 15 images of 3 independent experiments (I). (J-M) Human cervical tissue explants were inoculated with Pil+Opa_CEA_ GC with or without the SHP inhibitor NSC-87877 (20 μM) for 24 h. Shown are representative images (J). Arrowheads, shedding cells. Levels of epithelial shedding in the ectocervical (K), TZ (L), and endocervical (M) regions were quantified as above. Scale bar, 20 μm. *p<0.05; **p< 0.01; ***p<0.001, as determined by Student’s t-test and one-way non-parametric ANOVA (Kruskal-Wallis test).

Epithelial cell shedding is a mechanism by which the host removes colonizing bacteria. GC have been shown to inhibit epithelial shedding through CEACAM-dependent activation of integrin in human cell lines and CEACAM transgenic mice [28]. To examine the relationship between GC infectivity and shedding of the different epithelial cell types, we asked if GC cause epithelial shedding at each of the three cervical regions. By CFM, we observed that Pil+ GC, regardless of which Opa isoform was expressed or if Opa proteins were phase variable, induced epithelial shedding in all regions of the human cervix 24 h after inoculation, compared to no GC controls (Fig 5A, 5D and 5F). Multilayered ectocervical and TZ epithelial cells mostly shed in layers (Fig 5A and 5D), while monolayered endocervical epithelial cells shed individually (Fig 5F, arrowheads). We evaluated the levels of epithelial shedding in the ectocervix and TZ by measuring the thickness and the number of epithelial cell layers that remained in infected tissues relative to non-inoculation controls (Fig 5A–5E). The level of endocervical epithelial cell shedding was estimated by visual counting of the percentage of epithelial cells moving out of the epithelium (Fig 5F, arrowhead, and Fig 5G). Compared to no GC controls, the ectocervical and TZ epithelia in Pil+ΔOpa GC-inoculated tissue explants shed >60% and >50% of their thickness and cell layers, respectively (Fig 5A–5E), while the endocervix shed >30% of their epithelial cells (Fig 5F and 5G). As some of the exfoliated endocervical epithelial cells might be detached from the luminal surface, our method likely underestimated epithelial shedding in the endocervix. No significant epithelial shedding was observed in Pil- GC-infected cervical tissue explants. The expression of Opa_CEA_ or phase variable Opas (Opa+, wt MS11) but not Opa_HSPG_ significantly reduced epithelial cell shedding in the ecto/endocervix (Fig 5C and 5G). However, the expression of Opa_CEA_, Opa_HSPG,_ or phase variable Opas had no effect on the shedding of TZ epithelial cells (Fig 5E). The similar results from Opa+ GC- and Opa_CEA_ GC-infected cervical tissues are consistent with the notion that most of 11 Opa isoforms bind to CEACAMs. Thus, the expression of Opa_CEA_ or phase variable Opas, which enhances GC colonization of the ecto/endocervix and inhibits GC penetration into the endocervical subepithelium, suppresses GC-induced epithelial shedding in the two regions of the human cervix. The expression of Opa_CEA_, Opa_HSPG_, or phase variable Opas, which does not change GC infectivity in the TZ, also had no effect on GC-induced epithelial shedding.

To determine if CEACAM expression is required for the negative regulation of epithelial shedding by Opa_CEA_, we compared the shedding levels of HEC-1-B cells transiently transfected or not with CEACAM1L or 1S by measuring the percentage of cells that moved above non-polarized epithelial monolayers after GC inoculation (Fig 5H, arrowheads). The expression of either CEACAM1L or 1S did not significantly change the levels of HEC-1-B shedding induced by Pil+ΔOpa GC as well as the basal level of shedding (Fig 5H and 5I). However, the expression of CEACAM1L but not 1S significantly reduced the shedding of HEC-1-B cells infected with Pil+Opa_CEA_ GC back to basal levels (Fig 5H and 5I). We determined if SHP was involved by treating HEC-1-B cells and cervical explants with the SHP inhibitor NSC-87877 before and during GC inoculation. Treatment of the SHP inhibitor enhanced the shedding of Opa_CEA_ GC-infected HEC-1-B cells that expressed CEACAM1L (Fig 5H and 5I). Furthermore, SHP inhibition increased epithelial shedding of Opa_CEA_ GC-infected ecto/endocervical tissues to the level in ΔOpa GC-infected tissues but had no effect on epithelial shedding in the TZ (Fig 5J–5M). These data suggest that expression of CEACAM1L inhibits GC-induced epithelial exfoliation of the human cervix probably through interacting with Opa_CEA_ and activating downstream SHP. Together, these data suggest that GC can enhance cervical colonization or penetration by inhibiting or enhancing epithelial shedding, respectively.

### GC induce the disassembly of E-cadherin-based cell-cell junctions exclusively in the TZ and the endocervix

**Fig 6:**
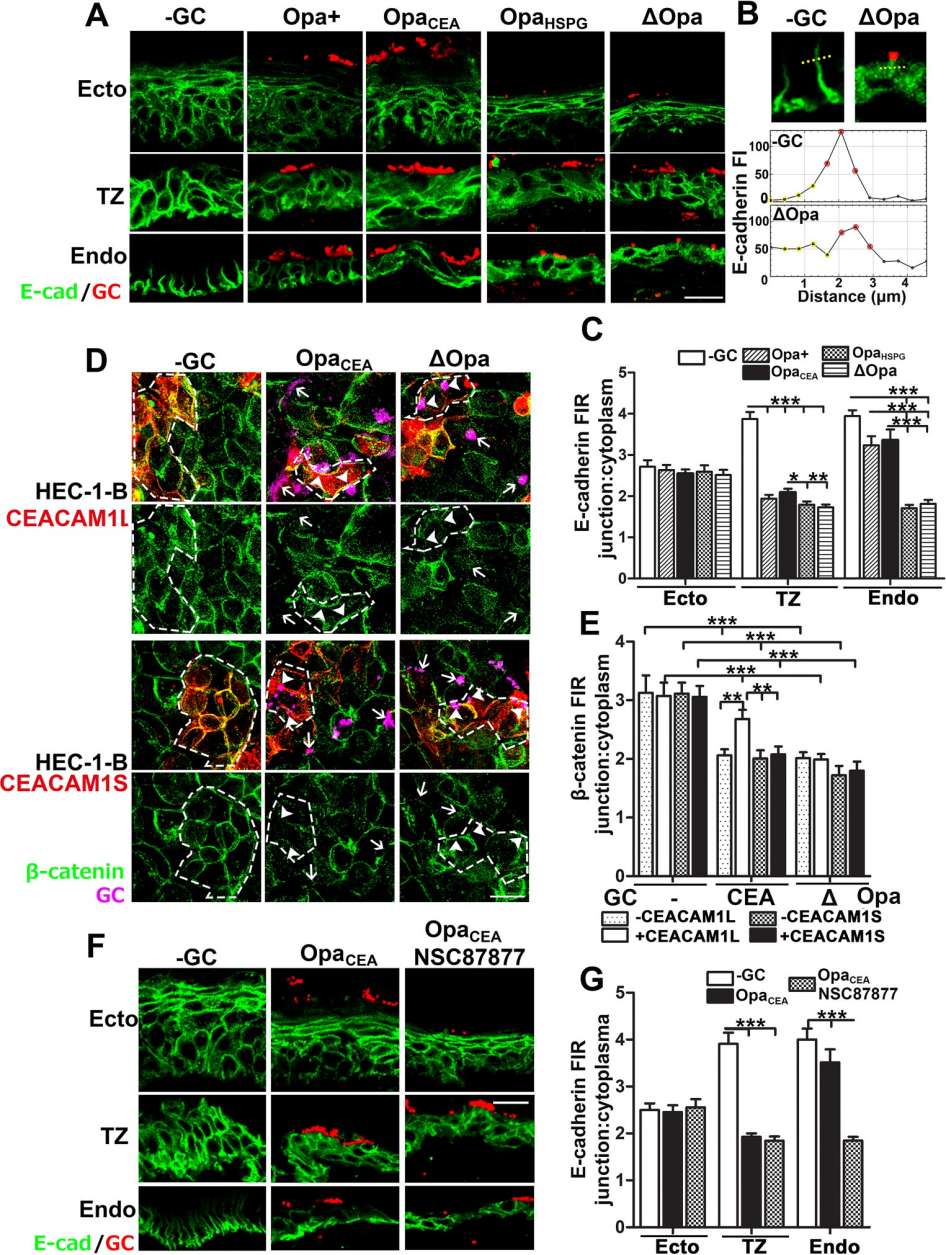
GC induce the disassembly of E-cadherin-based cell-cell junctions exclusively in the TZ and the endocervix. (A-C) Tissue explants were inoculated with Pil+ Opa_CEA_, Opa_HSPG_ or ΔOpa GC for 24 h (MOI~10), washed at 6 and 12 h post-inoculation, and cryopreserved. Tissue sections were collected, processed, stained for GC and E-cadherin (E-cad), and analyzed using CFM. Shown are representative CFM images (A). Disruption of cell-cell junctions was determined by the FI ratio (FIR) (±SEM) of E-cad (C) at the cell-cell border (red dots in B) relative to the cytoplasm (yellow dots in B) using FI line profiles crossing cell-cell junction (B). (D-E) HEC-1-B cells transfected with or without CEACAM1L or 1S, inoculated with Pil+Opa_CEA_ or Pil+ΔOpa GC for 6 h, and stained for GC, CEACAMs, and β-catenin. Shown are representative CFM images (D). Dashed lines, cells expressing CEACAM1L or 1S. Disruption of cell-cell junctions was determined by the FIR (±SEM) of β-catenin at the cell-cell border to the cytoplasm (E). (F-G) Human cervical tissue explants were inoculated with Pil+Opa_CEA_ GC with or without the SHP inhibitor (20 μM) for 24 h. Tissue sections were stained for E-cad and GC. Shown are representative CFM images (F). Disruption of cell-cell junctions was determined by the FIR (±SEM) of E-cad at the cell-cell border to the cytoplasm (G). Shown were the average of 50~200 individual cells in 20 randomly acquired images from each cervical region of 3 human cervixes (C and G) or 3 independent experiments (E). Scale bar, 20 μm. **p< 0.01; ***p<0.001, as determined by Student’s t-test and one-way non-parametric ANOVA (Kruskal-Wallis test).

We previously showed that GC disrupt the apical junction of endocervical epithelial cells to induce epithelial shedding and bacterial penetration [30]. To determine if varying GC infectivity is due to differential regulation of epithelial cell-cell junctions in the three cervical regions, we analyzed the integrity of E-cadherin (Ecad)-based cell-cell junctions, as they are shared by the epithelium of the three cervical regions. We used the translocation of E-cad from the cell-cell junction to the cytoplasm as an indicative of junction complex disassembly and quantified the disassembly by the fluorescence intensity ratio (FIR) of E-cad staining at the cell-cell junction relative to the cytoplasm (Fig 6A and 6B). In uninfected cervical tissue explants, E-cad staining concentrated at the epithelial cell-cell junction of all regions except for the luminal layer of ectocervical epithelial cells showing no staining (Fig 6A and S5 Fig). Inoculation of Pil+Opa+, Opa_CEA_, Opa_HSPG_ or ΔOpa GC all reduced the junction-to-cytoplasm FIR in TZ epithelial cells from ~4 to ≤2, while the E-cad FIR of endocervical epithelial cells was significantly reduced by Pil+Opa_HSPG_ and Pil+ΔOpa but not Pil+Opa+ and Opa_CEA_ GC, compared to no GC controls (Fig 6A–6C and S5 Fig). In contrast, the FIR of ectocervical epithelial cells, where no GC penetration was detected, was not affected by GC inoculation or by the Opa variant expressed, despite epithelial shedding (Fig 6C). These results indicate that GC induce disassembly of E-cad-based junctions in the TZ and the endocervix but not in the ectocervix. Expression of Opa_CEA_ or phase variable Opas only inhibits GC-induced disassembly of E-cad cell-cell junctions in the endocervix with high CEACAM expression levels but not in the TZ with very low CEACAM expression levels.

To determine if Opa_CEA_-CEACAM interaction inhibits GC effect on epithelial cell-cell junction, we compared the ability of GC to induce cell junction disassembly in HEC-1-B cells with or without expression of CEACAM1L or 1S. We analyzed junction disassembly in HEC-1-B cells by measuring the junction-to-cytoplasm FIR of β-catenin, a protein linking E-cad to cell signaling, the cytoskeleton, and CEACAMs [39, 40]. Inoculation of Pil+ΔOpa GC significantly reduced the junction-to-cytoplasm FIR of β-catenin in HEC-1-B cells, regardless of whether CEACAM1L or 1S was expressed or not (Fig 6D and 6E). However, inoculation of Pil+Opa_CEA_ only reduced the β-catenin FIR in CEACAM1S- but not CEACAM1L-expressing cells (Fig 6D and 6E). We further examined the involvement of SHP in regulating GC-induced disassembly of E-cad-based cell-cell junction in the cervix. Treatment with the SHP inhibitor reduced the junction-to-cytoplasm FIR of E-cad in endocervical epithelial cells inoculated with Pil+Opa_CEA_ GC, but did not affect the E-cad FIR in infected ectocervical and TZ epithelial cells (Fig 6F and 6G). Together, our data indicate that GC exclusively disrupt the E-cad-based cell-cell junction of the endocervix and the TZ, and Opa_CEA_-CEACAM interaction in the endocervix inhibits this disruption probably through downstream molecules, like SHP.

### GC differentially regulate β-catenin phosphorylation and integrin-β1 activation in various cervical epithelial cells

**Fig 7:**
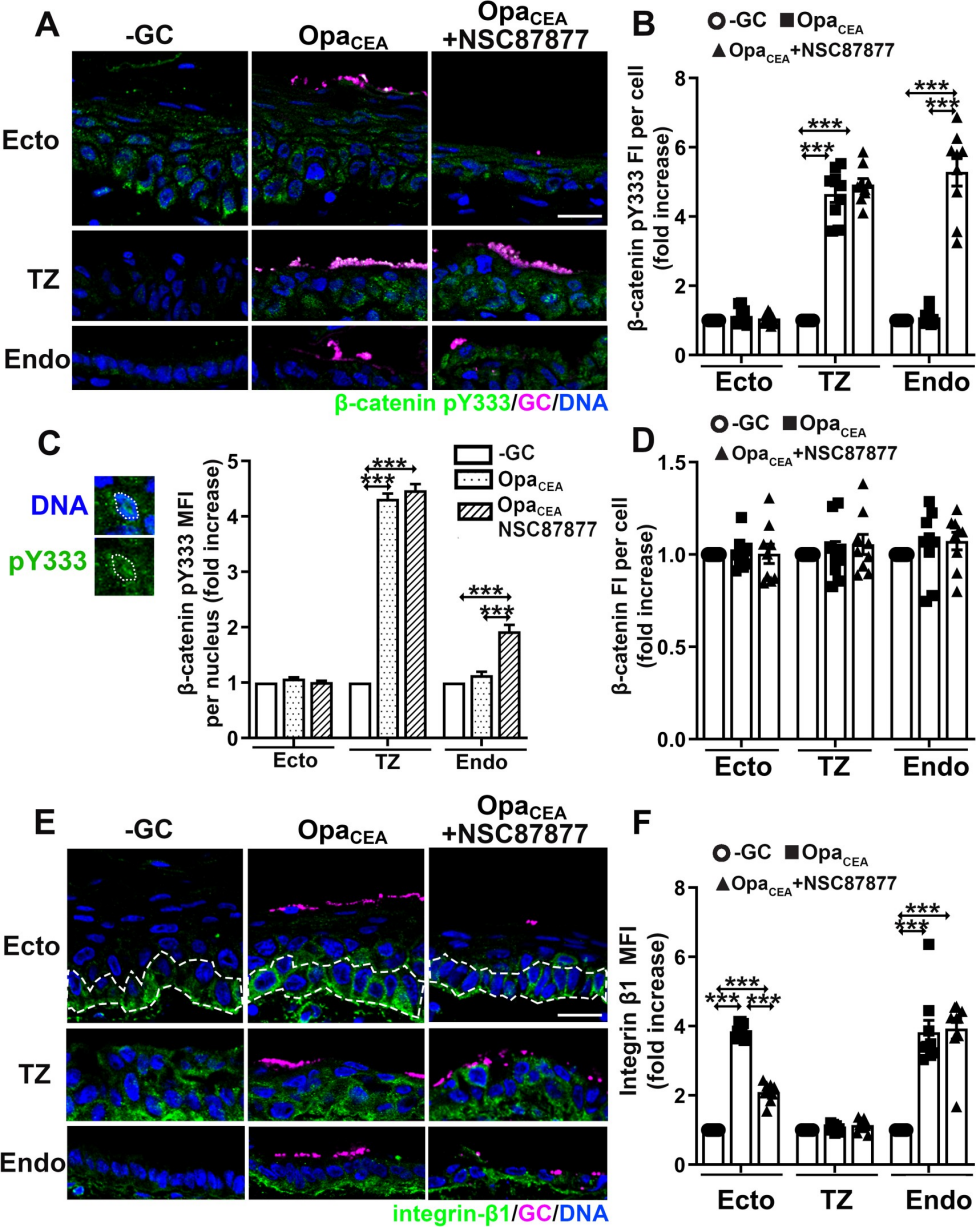
GC differentially regulate β-catenin phosphorylation and integrin β1 activation in various cervical epithelial cells. Cervical tissue explants treated with or without the SHP inhibitor, inoculated with or without Pil+Opa_CEA_ GC for 24 h. (A-D) Tissue sections were stained for β-catenin pY333 and total β-catenin proteins. (A) Shown are representative CFM images. (B) The levels of β-catenin pY333 were quantified using CFM images as folds of increases in its FI (±SEM) per epithelial cell compared to uninfected controls. Shown are the means from 9 images acquired for each cervical region of 3 human cervixes. (C) The levels of β-catenin pY333 in the nucleus were quantified using CFM images as folds of increases in its MFI (±SEM) per epithelial nucleus compared to uninfected controls. Shown are the means from ~400 cells in 9 images per cervical region of 3 human subjects. (D) The levels of β-catenin protein were quantified using CFM images as folds of increases in its FI (±SEM) per epithelial cell compared to uninfected controls. (E-F) Tissue sections were stained for active integrin β1. (E) Representative CFM images. (F) Levels of active integrin β1 were quantified using CFM images as folds of increases in its MFI (±SEM) in cervical epithelial cells (except for the basal layer in the ectocervix) compared to uninfected controls. Shown are the means from 9 images acquired for each cervical region of 3 human cervixes. Scale bar, 20 μm. ***p<0.001, as determined by Student’s t-test and one-way non-parametric ANOVA (Kruskal-Wallis test).

Intact E-cad cell-cell junctions in GC-inoculated ectocervical epithelial cells despite heavy shedding suggests that GC induce shedding of ectocervical epithelial cells through a mechanism different from GC-induced shedding of TZ and endocervical epithelial cells. To examine this, we evaluated the levels of the total β-catenin protein and phosphorylated β-catenin at Y333 (pY333) in various cervical epithelial cells that were inoculated with Pil+Opa_CEA_ GC and treated with or without the SHP inhibitor, as well as the level of β-catenin pY333 in the epithelial nuclei. The phosphorylation of β-catenin induces the disassociation of β-catenin from E-cad cell-cell junctions, leading to junction disassembly, and its dephosphorylation by tyrosine phosphatases, like SHP1/2, can stabilize the junction [41]. Phosphorylated β-catenin can translocate into the nuclei to regulate transcription [42]. Pil+Opa_CEA_ GC increased the FI of β-catenin pY333 >4 folds in individual TZ epithelial cells (Fig 7A and 7B) and their nuclei (Fig 7C) but not ecto/endocervical epithelial cells, compared to no GC controls. Treatment of the inhibitor specific for the tyrosine phosphatases SHP1/2 NSC87877, which increases GC penetration into the endocervical but not the TZ and ectocervical epithelium, increased the β-catenin pY333 FI in infected endocervical epithelial cells and their nuclei but not ectocervical and TZ cells (Fig 7A–7C). However, neither GC inoculation nor treatment of the SHP inhibitor changed the staining levels of the total β-catenin protein in epithelial cells of the three cervical regions (Fig 7D). These data indicate that GC induce β-catenin pY333 and its nuclear translocation in TZ and endocervical but not ectocervical epithelial cells. Opa_CEA_ expression inhibits and SHP inhibitor enhances β-catenin pY333 in the CEACAM-expressing endocervix but not the low CEACAM-expressing TZ.

Previous studies using a mouse model expressing human CEACAMs and human cell lines suggest the involvement of integrin-β1 in epithelial shedding and GC colonization [28]. We examined if integrin-β1 was differentially involved in GC infection in various cervical regions by immunofluorescence staining of integrin-β1 in its active conformation. Without GC inoculation, active integrin-β1 was detected in the basal layer of ectocervical epithelial cells, all TZ epithelial cells, and the basal membrane of endocervical epithelial cells (Fig 7E). Inoculation of Pil+Opa_CEA_ GC increased the MFI of active integrin-β1 ~4 folds in the upper layers of ectocervical epithelial cells and endocervical epithelial cells, but not TZ epithelial cells (Fig 7E and 7F), compared to no GC controls. SHP inhibitor treatment, which increased ecto/endocervical epithelial shedding, reduced the MFI of active integrin-β1 in the ectocervix to the basal level but did not change the MFI of active integrin-β1 in the endocervix and TZ (Fig 7E and 7F). Our results suggest that Opa_CEA_ GC activate integrin-β1 in epithelial cells of the ecto/endocervix but not the TZ, and GC-induced integrin-β1 activation in the ectocervix but not the endocervix is sensitive to SHP inhibitor. Together, these data suggest that GC primarily target integrin-β1 in the ectocervix but β-catenin in the TZ and endocervix to regulate the infectivity.

## Discussion

In this study, we provide evidence that human cervical tissue explants can be used as an effective model for mimicking GC infection *in vivo*. We show that when piliated GC encounter heterogeneous mucosal surfaces, they preferentially colonize the ectocervix and the TZ region. In contrast to this colonization pattern, GC selectively penetrate into the subepithelium of the TZ and the endocervix, with a higher penetration level in the TZ when compared to the endocervix. These results are consistent with clinical observations using gonorrhea patient biopsies which detected GC in the subepithelia of the TZ and endocervix but not the ectocervix [7]. As GC infect exclusively humans and initiate infection of the FRT from the cervix, cervical tissue explants provide an excellent *ex vivo* infection model for understanding the pathogenesis of GC as well as other STI pathogens and for testing novel agents for gonorrhea and STI prevention and treatment.

Our studies using tissue explants suggest that colonization of the luminal surface and penetration into the subepithelial tissue are the primary ways by which GC infect the human cervix. It remains technically difficult to distinguish between extracellular and intracellular GC in infected cervical tissues. However, we detected very few GC between the luminal and basal surfaces of epithelial cells in cervical explants, which argues against GC invasion into and replication within individual epithelial cells as a significant route of dissemination. Even though GC-epithelial interactions may induce neutrophil infiltration, GC penetration is more likely to exposes the bacteria to immune cells in the subepithelium than colonization. Therefore, GC penetration is likely to induce inflammation, leading to symptomatic infection; and colonization of the mucosal surface may only cause local asymptomatic infection.

A major finding of this study is that differences in the properties of cervical epithelial cells are critical factors controlling potential infection outcomes, including cell-cell junctions holding epithelial cells together as a physical barrier and the expression of host cell receptors for GC. Our study reveals that epithelial cells in the three cervical regions not only express different levels but also exhibit a distinct cellular distribution of CEACAMs, extending beyond a recent report on differential expression of CEACAM isoforms in various parts of the FRT [22]. The high level of CEACAM expression on the luminal surface of the ectocervix drives strong colonization of Opa_CEA_ GC. The low expression level and intracellular location of CEACAMs in TZ epithelial cells abolish the effects of Opa_CEA_, enabling GC penetration regardless of which Opa variant GC express, explaining why subepithelial GC were found in the TZ of patients. These data suggest that GC infectivity may not only vary along the mucosal surface of the cervix but also with the menstrual cycle, pregnancy, and age, as these factors can change the expression levels of host receptors for GC and the size of the TZ [43]. Conversely, expression of host receptors may drive the selection of GC with particular variants of the surface molecules that can specifically bind to these host receptors. This is supported by recent studies using bioinformatic or functional analysis of human isolates that identified a positive selection pressure driving variation in the extracellular regions of GC surface proteins [44, 45].

Cell-cell junction complexes that seal the paracellular space are essential for the barrier function of the epithelium, which prevents pathogens from entering tissues. GC penetrate into endocervical tissues by disrupting the apical junction [30]. While heterogeneous cervical epithelial cells share E-cad-based junctions as the adherens junction and a part of the apical junction, this study shows that GC differentially regulate this junction complex along the cervix. GC selectively disrupt E-cad junctions in the TZ and the endocervix where GC can penetrate, but not in the ectocervix where GC cannot penetrate. Furthermore, GC only activate the phosphorylation of β-catenin (Y333), which leads to E-cad junction disassembly, in the TZ and endocervical but not ectocervical epithelial cells. These data provide evidence for epithelial cell-cell junction disruption as a mechanism for GC penetration. We observed that E-cad expression is uniquely absent in the luminal layers of ectocervical epithelial cells, even after GC-induced epithelial shedding. Consequently, GC have little chance to interact with ectocervical epithelial cells expressing E-cad directly, providing an explanation as to why GC fail to disrupt E-cad cell junctions in the ectocervix.

Epithelial shedding negatively regulates GC colonization. Previous studies show that GC can modulate shedding of vaginal and ectocervical epithelial cells of mice and nonpolarized human epithelial cells by targeting integrin β1 [46], which belongs to a family of proteins mediating cell adhesion to the extracellular matrix [47]. We extend this finding by showing that GC selectively regulate the activity of integrin β1 in ectocervical epithelial cells to control epithelial shedding. Differing from the ectocervix, GC induce shedding of TZ and endocervical epithelial cells by disrupting E-cad junctions. These results together shed critical lights on the mechanism by which distinct properties of cervical epithelial cells modulate GC infectivity.

While the roles of pili and Opa, two major surface molecules on GC, in GC infection have been studied extensively [13, 16, 21, 48–51], this study has defined a role for pili and Opa in GC infection of the human cervix *in vivo*. Our results show that pili are essential for GC to colonize the mucosal surface of all the three cervical regions, indicating an essential role for pili in initiating GC infection in the FRT. In contrast, Opa is not essential for either GC colonization or penetration as a Pil+ GC strain with all 11 Opa isoforms deleted can still colonize the cervix and penetrate the TZ and endocervical epithelia. However, the phase variation of Opa can change GC infection from favoring colonization to tissue penetration. The expression of Opa_CEA_ significantly increases GC colonization of the ecto/endocervical epithelia, but drastically inhibits GC penetration into the endocervical subepithelium (the bacteria do not penetrate into ectocervix regardless of Opa expression). These data suggest that turning on of Opa_CEA_ expression may limit GC infection in the human cervix to colonization, likely leading to asymptomatic infection. As the majority of the 11 Opa isoforms bind to CEACAMs and most isolates from patients [45, 52] and mouse infection models are Opa+ [48, 53], our results may explain why the percentage of asymptomatic GC infections is high in women.

Previous studies have shown a role for Opa proteins, particularly Opa_CEA_, in GC invasion into epithelial cells [18, 23]. Unfortunately, we cannot examine GC invasion into epithelial cells in tissue explants, due to technical difficulties in precisely distinguishing intracellular and extracellular bacteria using CFM. However, as discussed above, only limited GC staining was detected between the luminal and basal surfaces of the human cervical epithelia. This implies that GC invasion into and transcytosis through epithelial cells in the epithelium are not the dominant events for GC infection in the human cervix. Interestingly, our preliminary studies found GC inside exfoliating epithelial cells. Our observations on the absence of GC inside the cells of the epithelium and the presence of GC inside exfoliated epithelial cells are consistent with early clinical observations [7, 54]. However, whether Opas play a role in this invasion event as previously reported and the relationship of this invasion event with epithelial exfoliation are the interest of our future study.

This study provides direct evidence from both tissue explants and HEC-1-B cells that do and do not express CEACAMs that Opa_CEA_ modulates GC infection in cervical explants through CEACAMs, supporting the findings from CEACAM transgenic mice [22]. We further show here that SHP activation is a potential mechanism by which Opa_CEA_/CEACAM regulates GC infectivity, as an inhibitor specific for the enzymatic activity of SHP1/2 abolishes all the effects of Opa_CEA_ or CEACAM1L expression that we measured. However, our study does not exclude the possibility of additional mechanisms downstream CEACAMs.

**Fig 8:**
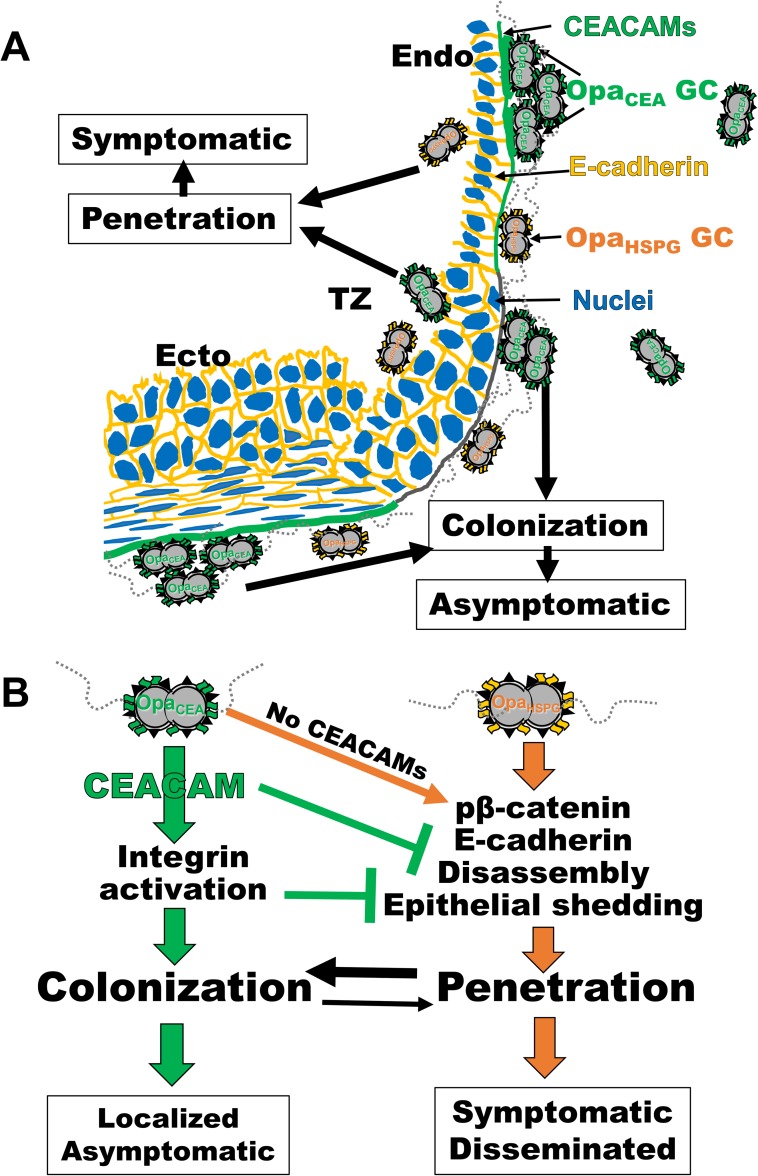
Both the heterogeneous properties of cervical epithelial cells and the surface molecules of GC regulate GC infectivity. The results of this study and numerous published studies enable us to propose a working model for GC pathogenesis in the FRT. (A) GC delivered into the vagina establish efficient and persistent colonization at the ectocervix and probably also at the TZ and endocervix through pili and evolution-driven expression of Opa_CEA_, which leads to asymptomatic local infections. GC can take advantage of the low CEACAM expression in the TZ to penetrate the epithelium, leading to cervicitis. Low expression of Opa_CEA_ allows GC to effectively penetrate into the endocervical epithelium, potentially causing symptomatic infection. (B) GC penetrate the epithelia of the endocervix and TZ by inducing the phosphorylation of β-catenin, which leads to the disassembly of E-cad-based epithelial cell-cell junctions and epithelial exfoliation. Opa-CEACAM interaction drives GC infectivity into the colonization mode while inhibiting the penetration mode, by blocking β-catenin phosphorylation and epithelial cell-cell junction disassembly and activating integrin-mediated epithelial adhesion, which reduce the shedding of GC-associated epithelial cells. Because GC with low levels of Opa_CEA_ expression are rare, as most 11 Opa proteins are Opa_CEA_, the majority of the bacteria colonize rather than penetrate the cervical epithelium, explaining why most infections of the FRT are asymptomatic and the invasive disease is rare.

Our findings that GC infectivity varies depending on the characteristics of epithelial cells with which GC interact and variants of GC surface molecules reconcile controversial results published in the field by explaining why different results were generated from different experimental systems. Combining the results of this study and numerous published studies, we propose the following working model for GC pathogenesis in the FRT (Fig 8). GC delivered into the vagina establish efficient and persistent colonization at the ectocervix and probably also at the TZ and endocervix through pili and evolution-driven expression of Opa_CEA_, which leads to asymptomatic local infections. GC can take advantage of the low CEACAM expression in the TZ to penetrate the epithelium, leading to cervicitis (Fig 8A). This penetration may also be a mechanism for GC to overcome the cervical mucus plug and epithelial shedding. Low expression of Opa_CEA_ allows GC to effectively penetrate into the endocervical epithelium by inducing the disassembly of epithelial cell-cell junctions, potentially causing symptomatic infection (Fig 8B). Opa-CEACAM interaction drives GC infectivity into the colonization mode while inhibiting the penetration mode, by blocking epithelial cell-cell junction disassembly and activating integrin-mediated epithelial adhesion, which reduce the shedding of GC-associated epithelial cells (Fig 8B). Because GC with low levels of Opa_CEA_ expression are rare, as most 11 Opa proteins are Opa_CEA_, this model provides a mechanistic explanation as to why most infections of the FRT are asymptomatic and why the invasive disease is rare.

## Materials and methods

### Neisseria strains

*N*. *gonorrhoeae* strain MS11 expressing both pili and Opa (MS11Pil+Opa+) was obtained from Dr. Herman Schneider, Walter Reed Army Institute for Research. Isogenic derivatives of this strain, MS11ΔOpa (all 11 *opa* genes deleted), MS11OpaH (CEACAM-binding, Opa_CEA_), and MS11OpaC (HSPG-binding, Opa_HSPG_) have previously been described [33, 34]. Isogenic strains generated from MS11Opa+, including OpaH, OpaC, and ΔOpa strains, express LOS structures that are similar to the parent strain [34]. MS11 Pil+Opa+ and Pil-Opa+ GC were identified based on the morphology of their colonies using a dissecting light microscope. Our previous sequencing analysis showed that they express different *pilE* variants [34]. GC were grown on plates with GC media (Difco, BD Bioscience) and 1% Kellogg’s supplement [55] for 16–18 h before inoculation. The concentration of GC in suspension was determined using a spectrophotometer and inoculated at an MOI around 1:10, one luminal cervical epithelial cell to 10 bacteria.

### Human cervical tissue explants

The tissue explants were cultured as previously described [31]. Cervical tissues were obtained from patients undergoing voluntary hysterectomies and received within 24 h post-surgery. Samples were cut into ~2.5 cm (L) X 0.6 cm (W) X 0.3 cm (H) pieces, incubated in CMRL-1066 (GIBCO) plus antibiotics for 24 h and then in antibiotic-free media for another 24 h, before inoculation with GC as previously described [56]. The number of luminal cervical epithelial cells in each tissue explants was estimated by dividing the luminal surface area of a tissue explant by the average luminal surface area of individual epithelial cells (25 μm^2^). The luminal surface area of each tissue explant was measured using microscopic images and NIH ImageJ.

### Immunofluorescence analysis of human cervical tissue explants

Individual cervical tissue pieces were inoculated with GC at an MOI of ~10 and incubated for 24 h. When indicated, tissue pieces were inoculated with GC in the presence or absence of the SHP inhibitor NSC87877 (20 μM, EMD Millipore). Unassociated GC were removed by extensive washes at 6 and 12 h post-inoculation. The tissue was then fixed, embedded in gelatin, cryopreserved, sectioned crossing the luminal and basal surfaces of the epithelium, stained for F-actin (Cytoskeleton), E-cadherin (BD Bioscience), CEACAMs (monoclone antibody YTH71.3, cross-react with CEACAM1, 3, and 6, Santa Cruz Biotechnology), β-catenin (EMD Millipore), phosphorylated β-catenin (Y333) (Thermo Fisher Scientific), integrin β1 in the active conformation (monoclonal antibody 9EG7, BD Bioscience), and GC [57] by specific antibodies, and nuclei by Hoechst (Life Technologies), and imaged using 40X objective on a confocal fluorescence microscope (Zeiss LSM 710, Carl Zeiss Microscopy LLC) as previously described [56]. Images were randomly acquired from the ectocervix to the endocervix as single images or Z-series of 0.57 μm/image, and 3D composites obtained using Zeiss Zen software.

Levels of GC colonization were quantified by two methods using confocal images: (1) the percentage of GC-associated luminal epithelial cells versus the total number of luminal epithelial cells by visually accounting, and (2) the average fluorescence intensity (FI) of GC staining per μm^2^ of the luminal surface using the NIH ImageJ software. The data were generated using CFM images from three independent analyses of each of three human cervixes. Levels of GC penetration were also determined by two methods: (1) the percentage of epithelial cells with GC staining at and below the basal membrane verses of the total number of GC-associated epithelial cells through visually counting, and (2) the percentage of the FI of GC staining at subepithelial tissue versus the total FI of GC staining at both the subepithelium and the luminal surface in each randomly acquired image. The data were generated using 12~18 CFM images per cervical region from two to three independent analyses of each of three human cervixes.

Levels of CEACAM expression in epithelial cells were measured by FI of CEACAM staining per cell. The data were generated using 21 CFM images per cervical region acquired from 2~4 independent analyses of three human cervixes.

The recruitment of CEACAMs to GC was evaluated by the percentage of GC microcolonies with an accumulation of CEACAM staining underneath or nearby versus the total number of GC microcolony through FI profiles of GC and CEACAM staining at the luminal surface of cervical epithelia. The data were generated using images from two independent analyses of each of three human cervixes.

Levels of epithelial exfoliation in the ectocervix and the transformation zone (TZ) was determined by two methods: (1) the percentage of the remaining thickness (μm) of the epithelium in infected versus uninfected ectocervical and TZ tissue explants, and (2) the percentage of remaining epithelial cell layers in infected versus uninfected ectocervical and TZ tissue explants. Levels of epithelial exfoliation in the endocervix were determined by the percentage of epithelial cells localized on the top of the epithelial monolayer versus the total number of epithelial cells through visual inspection. The data were generated using 12~30 CFM images acquired per cervical region from 2~3 independent analyses of each of three human cervixes.

The redistribution of E-cadherin from the cell-cell junction to the cytoplasm was evaluated by the fluorescence intensity ratios (FIR) of E-cadherin staining at the cell-cell junction versus that in the cytoplasm in individual epithelial cells using CFM images and the NIH ImageJ software. The data were generated using 50~200 individual epithelial cells per cervical region from 20 randomly acquired CFM images of three human cervixes.

The levels of phosphorylated β-catenin at Y333 and total β-catenin were quantified as folds of increases in the FI of the immunofluorescence staining per epithelial cell in inoculated cervical explants, compared to no GC controls. The levels of active integrin β1 were quantified as folds of increases in the MFI of the immunofluorescence staining in the epithelium. For the ectocervix, the basal layer of epithelial cells, which exhibited strong staining for active integrin β1 in both GC inoculated tissue explants and no GC controls, were excluded from the quantification. The data were generated using nine CFM images per cervical region acquired from three human cervixes. The levels of phosphorylated β-catenin in epithelial nuclei were evaluated by folds of increase in the MFI of the staining in individual nuclei indicated by Hoechst staining, compared to no GC controls. The data were generated based on ~400 cells per cervical region in nine CFM images acquired from three human cervixes.

### Epithelial cells and CEACAM1 transfection

HEC-1-B, a human endometrial adenocarcinoma cell line (ATCC), was maintained in Eagles MEM alpha medium supplemented with 10% heat-inactivated FBS. Cells were seeded at 3.5 x10^5^ per dish (60 mm diameter, Thermo Fisher Scientific) and cultured for two days before transfection. Cells were transfected using 5 μg of a plasmid containing CEACAM1L or CEACAM1S cDNA and lipofectamine 3000 reagents (Thermo Fisher Scientific). Transfected cells were seeded at 6x10^4^ per transwell (6.5 mm diameter and 3 μm pore size, Corning) and cultured for two days before inoculation with GC.

T84, a human colorectal carcinoma cell line (ATCC), was maintained in DMEM:Ham F12 (1:1) supplemented with 7% heat-inactivated fetal bovine serum (FBS). Cells were seeded at 6x10^4^ per transwell (6.5 mm diameter and 3 μm pore size, Corning) and cultured for ~10 days until transepithelial electrical resistance (TEER) reached >2000 Ω before GC inoculation. TEER was measured using a Millicell ERS volt-ohm meter (EMD Millipore).

### Immunofluorescence analysis of epithelial cells

HEC-1-B cells, two days post-transfection, were inoculated with GC at an MOI of 10 for 6 h. Cells were washed and fixed with 4% paraformaldehyde, permeabilized with 0.1% Triton X100, and stained with anti-E-cadherin (BD Bioscience), anti-β-catenin (EMD Millipore), anti-CEACAMs (Santa Cruz Biotechnology), anti-GC antibodies, and Hoechst for nuclei. Series of z-images were randomly acquired using CFM to generate maximal projections. Levels of GC adherence to CEACAM-expressing and non-expressing cells were compared by the mean fluorescence intensity (MFI) ratio of GC staining in CEACAM-positive cells versus CEACAM-negative cells in the same size of ROIs in the same image. Epithelial exfoliation was quantified using xz images by the percentage of HEC-1-B cells moving above the monolayer. The redistribution of β-catenin from the cell-cell junction to the cytoplasm was quantified using the FIR of β-catenin staining at the cell-cell junction versus the cytoplasm as described for the E-cadherin in tissue explants. The results were generated from three independent experiments and five randomly acquired images from each condition in each experiment.

### GC transmigration assays

The assays were performed as previously described [57]. Briefly, polarized T84 epithelial cells that were pretreated with or without the SHP inhibitor NSC87877 (20 μM, EMD Millipore) for 1 h were incubated apically with GC (MOI = 10) at 37°C for 6 h. The basolateral media were collected and cultured, and the resulting colonies were counted as transmigrated bacteria.

### Statistical analysis

Statistical significance was assessed using the Student’s t-test and one-way non-parametric ANOVA (Kruskal-Wallis test) by Prism software (GraphPad Software).

### Ethics statement

Human cervical tissue was obtained from the National Disease Research Interchange (NDRI, Philadelphia, PA). Human cervical tissues used were anonymized. The use of human tissues for this research has been approved by the Institution Review Board of the University of Maryland.

## Supporting information

S1 Fig Human cervical tissue explants maintain the *in vivo* characteristics in culture. Human cervical tissue explants were cultured for three days and cryopreserved. Tissue sections were collected across the luminal and basal surface of epithelia, stained for DNA, E-cadherin, cytokeratin and/or F-actin, and analyzed using CFM. (A) Representative images of the mucosal epithelial regions of cervical tissue explants combining >30 images acquired using 10X objective. Dashed lines indicate the boundary between the endocervix and the TZ and between the TZ and the ectocervix. Scale bar, 100 μm. (B) Representative images of the three regions of cervical tissue explants. Scale bar, 20 μm.
(TIF)

S2 Fig Confocal fluorescence microscopic images of cervical tissue explants inoculated with or without GC. Human cervical tissue explants were incubated with MS11 Pil+Opa+ GC (Opa+) for 24 h, washed at 6 and 12 h to remove unassociated GC, and cryopreserved. Tissue sections were collected across the luminal and basal surface of epithelia and stained for GC, DNA, and F-actin. Images were acquired using 40X objective by a confocal fluorescence microscope (CFM, Zeiss LSM710). Shown are representative uncropped images from three cervical regions of human tissue explants that were inoculated with or without GC (-GC). Scale bar, 20 μm.
(TIF)

S3 Fig Treatment of the SHP inhibitor NSC-87877 has no significant effect on GC growth. MS11 Pil+Opa_CEA_ was cultured in GC media (with 1% Kellogg’s supplement and 1% NaHCO_3_) in the absence or presence of NSC-87877 (20 μM). The bacterial CFU was numerated at 6, 12 and 24 h. Shown are average CFU (±SEM) of three independent experiments.
(TIF)

S4 Fig Treatment of the SHP inhibitor increases Pil+Opa_CEA_ but not Pil+ΔOpa GC transmigration across polarized colonic epithelial cells. The transmigration of Pil+Opa_CEA_ and Pil+ΔOpa GC across polarized T84 epithelial cells treated with or without the SHP inhibitor (20 μM) is showed as the fold of the increase in GC CFU in the basal medium compared to the CFU of transmigrated Pil+Opa_CEA_ GC without SHP inhibitor treatment. Shown are average CFU (±SEM) of three independent experiments.
(TIF)

S5 Fig GC inoculation disrupts E-cadherin-based cell-cell junction. Representative 3D images of the TZ and endocervical epithelium in human cervical tissue explants that were inoculated with or without Pil+Opa_CEA_ or Pil+ΔOpa GC and stained for GC and E-cadherin. Scale bar, 20 μm.
(TIF)

S1 Video Three-dimensional images of human cervical tissue sections. Human cervical tissue explants were cultured for three days and cryopreserved. Tissue sections were collected across the luminal and basal surface of epithelia, stained for DNA, E-cadherin, and F-actin, and analyzed using CFM and Zen software. Shown are representative 3D images of the epithelia of the ectocervical, TZ, and endocervical regions.
(MP4)

S2 Video Penetration of Pil+Opa_CEA_ GC into the subepithelium of the TZ. Human cervical tissue explants were inoculated with Pil+Opa_CEA_ GC for 24 h. Thin sections of infected tissue explants were stained for DNA, F-actin, and GC and analyzed using CFM and Zen software. Shown are representative 3D images of the epithelia of the ectocervical, TZ, and endocervical regions. Arrows, GC penetrated into the subepithelium.
(MP4)

S3 Video Distribution of CEACAMs in the human cervical tissue. Thin sections of human cervical tissue explants were stained for DNA and CEACAMs and analyzed using CFM and Zen software. Shown are representative 3D images of the epithelia of the ectocervical, TZ, and endocervical regions.
(MP4)

S4 Video CEACAMs are recruited to the adherent sites of Pil+Opa_CEA_ GC on the endocervical but not ectocervical and TZ epithelial cells. Human cervical tissue explants were inoculated with Pil+Opa_CEA_ GC for 24 h. Thin sections of infected tissue explants were stained for DNA, CEACAMs, and GC and analyzed using CFM and Zen software. Shown are representative 3D images of the epithelia of the ectocervical, TZ, and endocervical regions. Arrows, GC microcolonies recruiting CEACAMs.
(MP4)
