# Supplementary figures and images for: Neisseria gonorrhoeae infects the heterogeneous epithelia of the human cervix using distinct mechanisms
Source: PLoS Pathog. 2019 Dec 2;15(12):e1008136. doi: 10.1371/journal.ppat.1008136 (PMC6907876; doi:10.1371/journal.ppat.1008136)

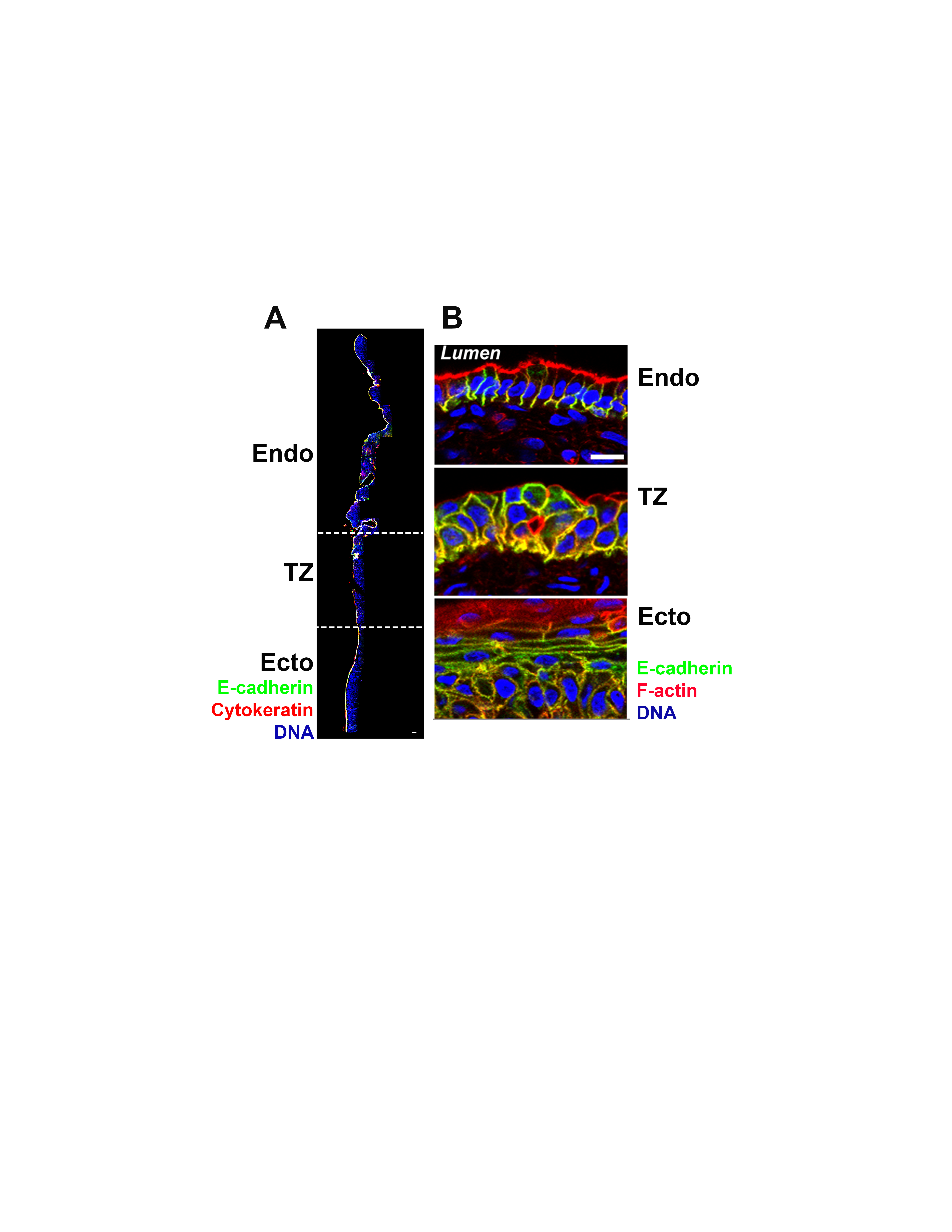

Supplement: S1 Fig — Human cervical tissue explants were cultured for three days and cryopreserved. Tissue sections were collected across the luminal and basal surface of epithelia, stained for DNA, E-cadherin, cytokeratin and/or F-actin, and analyzed using CFM. (A) Representative images of the mucosal epithelial regions of cervical tissue explants combining >30 images acquired using 10X objective. Dashed lines indicate the boundary between the endocervix and the TZ and between the TZ and the ectocervix. Scale bar, 100 μm. (B) Representative images of the three regions of cervical tissue explants. Scale bar, 20 μm. (TIF) [file ppat.1008136.s001.tif]

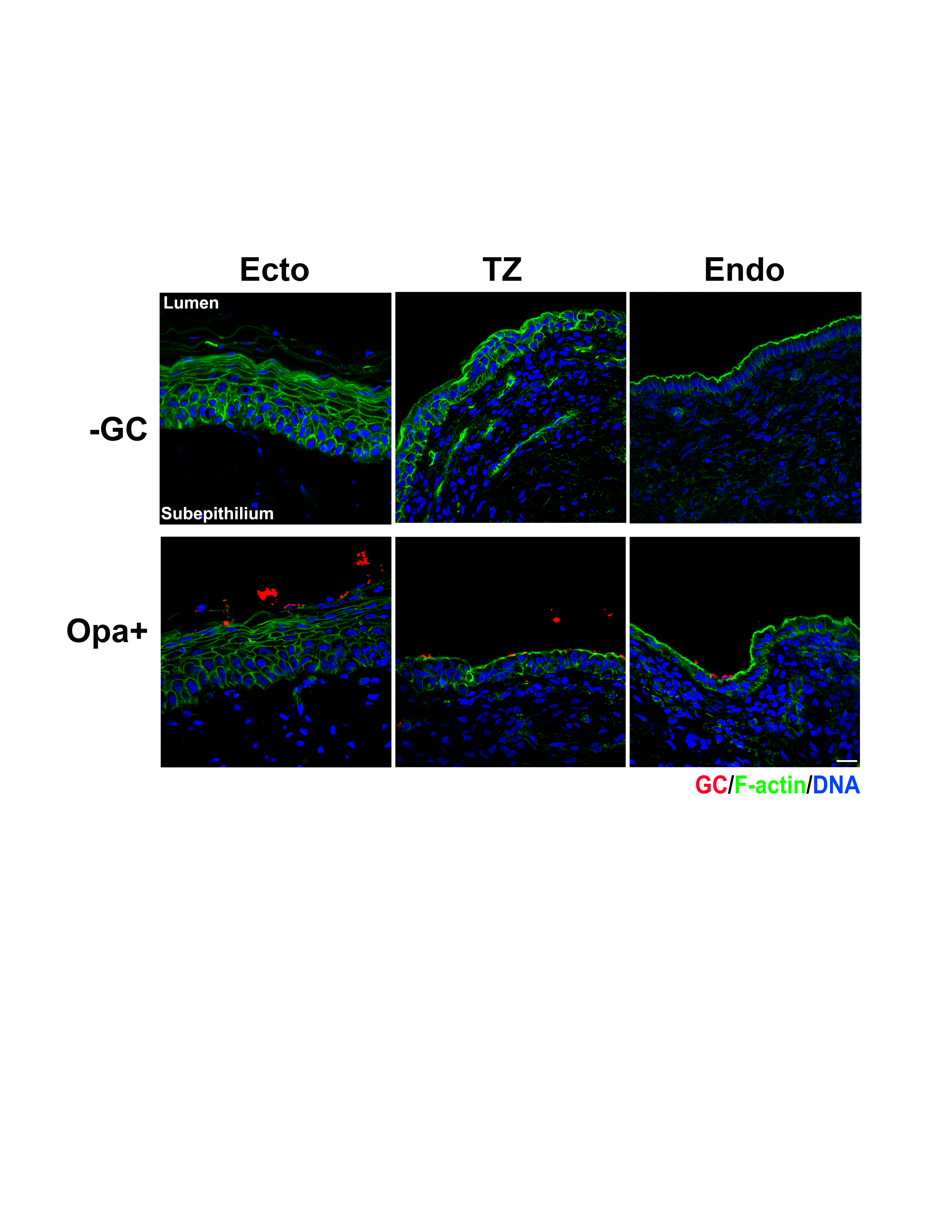

Supplement: S2 Fig — Human cervical tissue explants were incubated with MS11 Pil+Opa+ GC (Opa+) for 24 h, washed at 6 and 12 h to remove unassociated GC, and cryopreserved. Tissue sections were collected across the luminal and basal surface of epithelia and stained for GC, DNA, and F-actin. Images were acquired using 40X objective by a confocal fluorescence microscope (CFM, Zeiss LSM710). Shown are representative uncropped images from three cervical regions of human tissue explants that were inoculated with or without GC (-GC). Scale bar, 20 μm. (TIF) [file ppat.1008136.s002.tif]

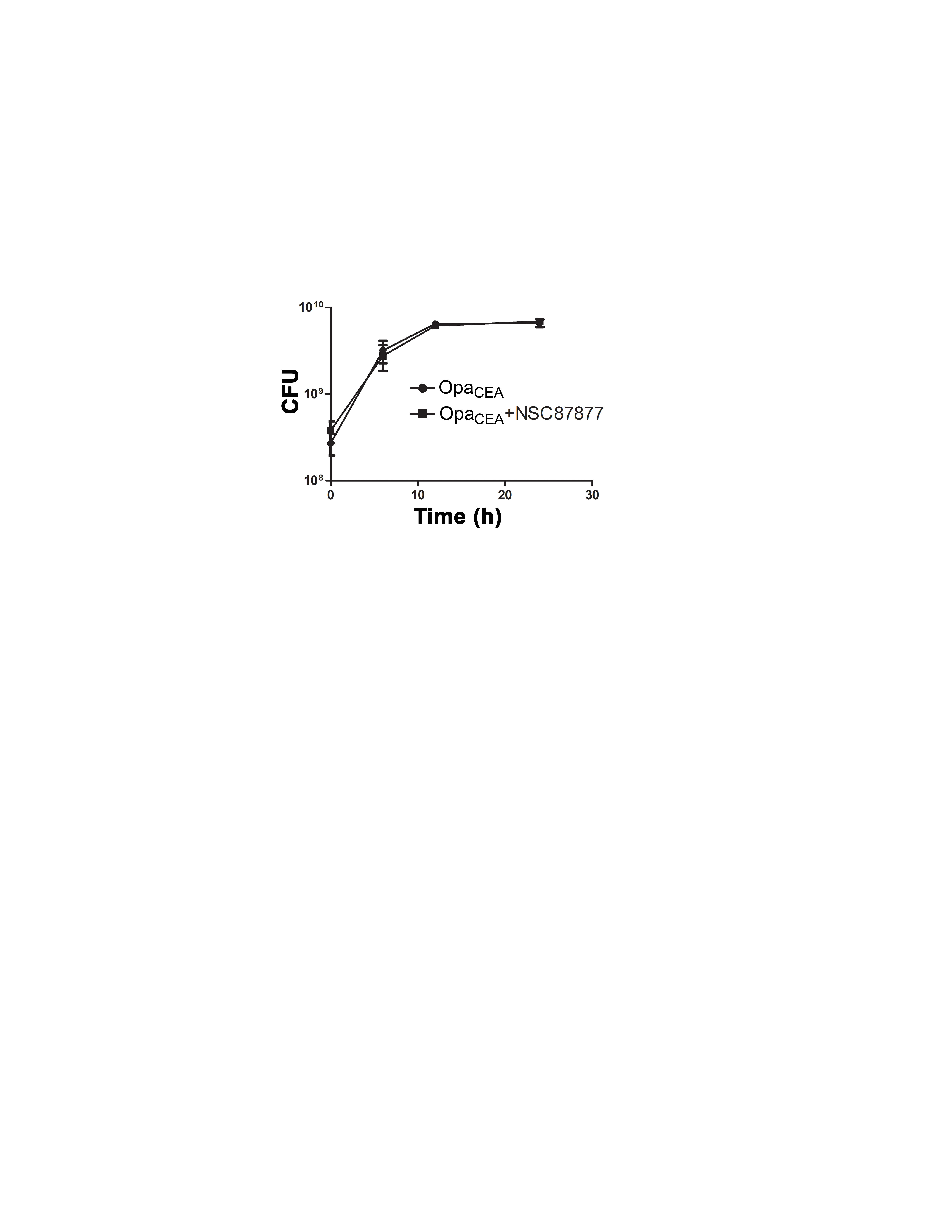

Supplement: S3 Fig — MS11 Pil+OpaCEA was cultured in GC media (with 1% Kellogg’s supplement and 1% NaHCO3) in the absence or presence of NSC-87877 (20 μM). The bacterial CFU was numerated at 6, 12 and 24 h. Shown are average CFU (±SEM) of three independent experiments. (TIF) [file ppat.1008136.s003.tif]

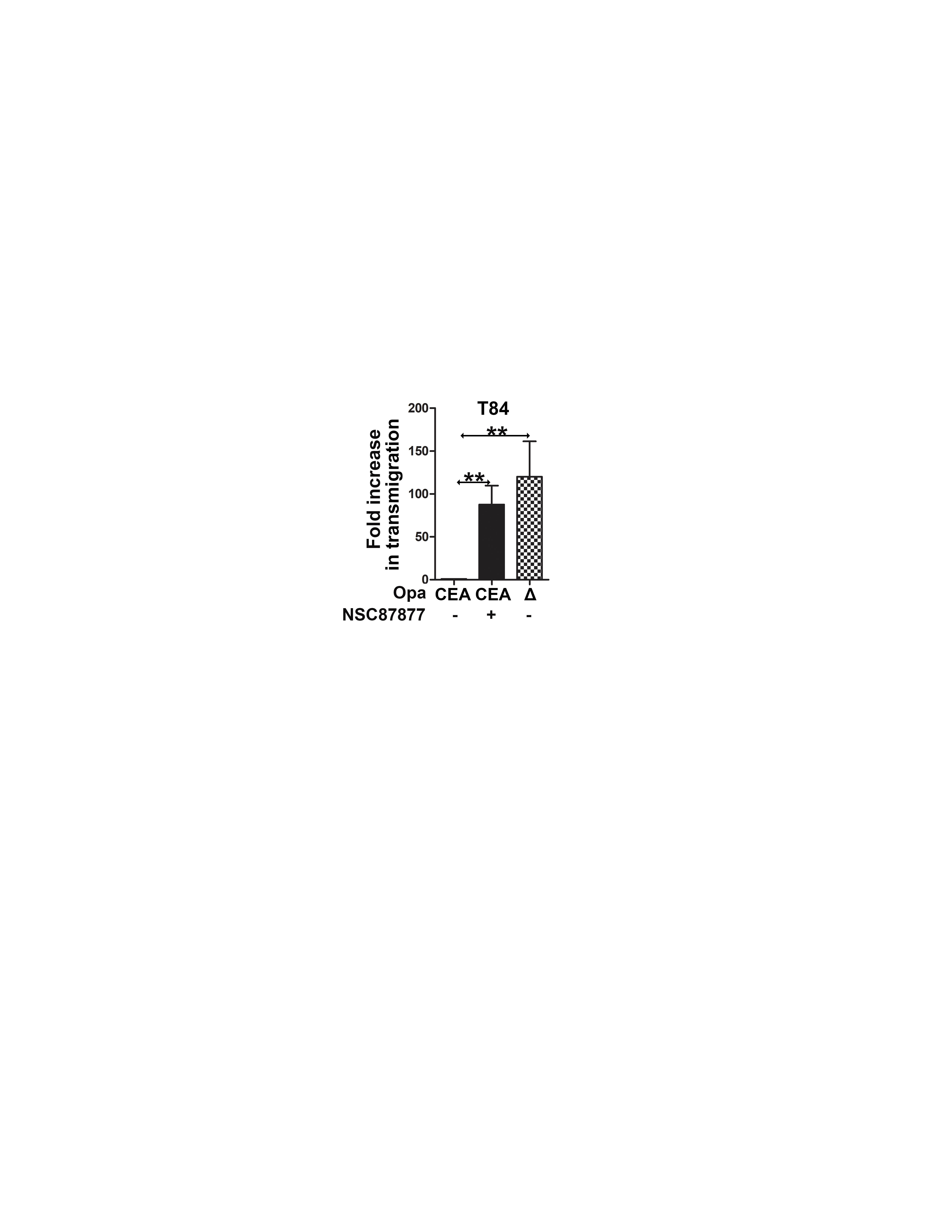

Supplement: S4 Fig — The transmigration of Pil+OpaCEA and Pil+ΔOpa GC across polarized T84 epithelial cells treated with or without the SHP inhibitor (20 μM) is showed as the fold of the increase in GC CFU in the basal medium compared to the CFU of transmigrated Pil+OpaCEA GC without SHP inhibitor treatment. Shown are average CFU (±SEM) of three independent experiments. (TIF) [file ppat.1008136.s004.tif]

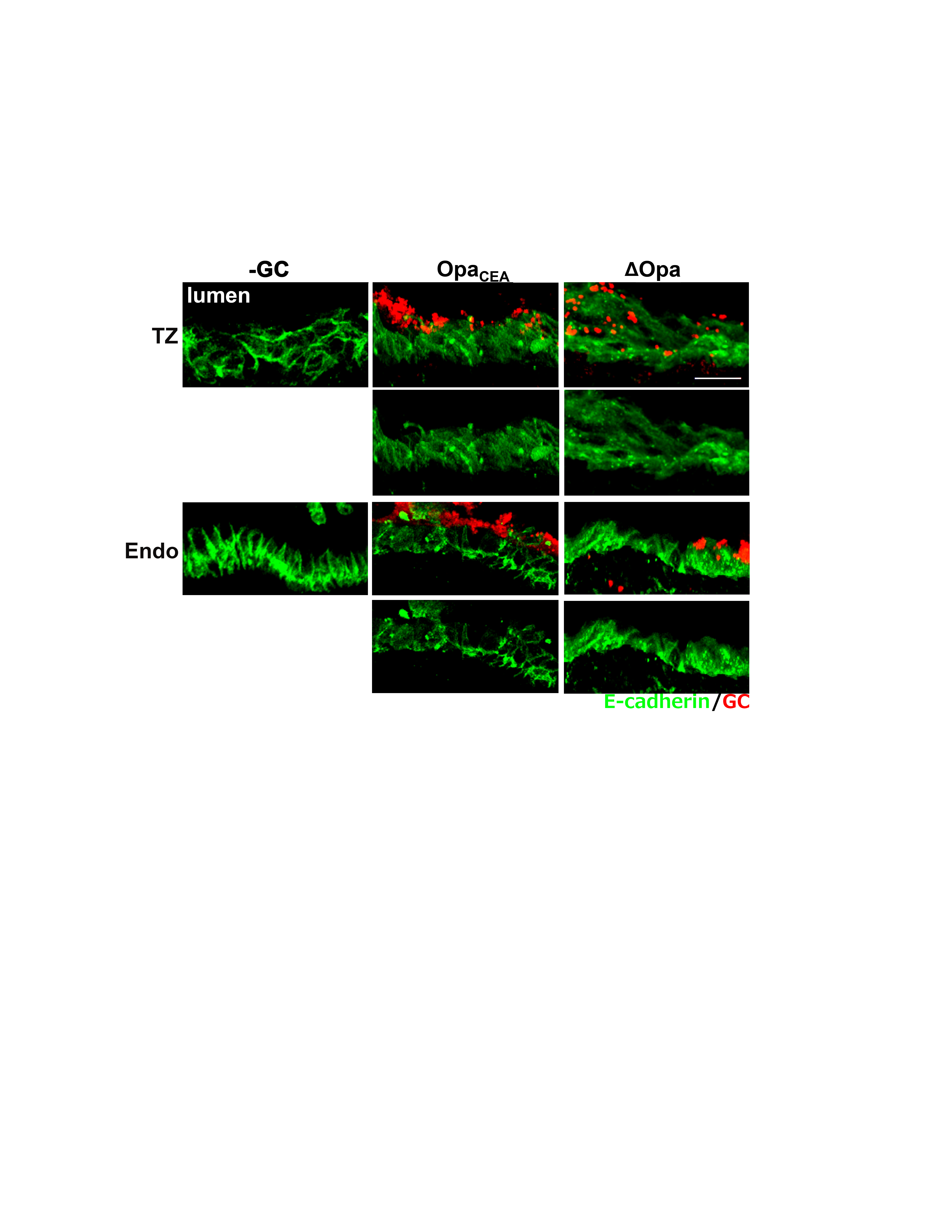

Supplement: S5 Fig — Representative 3D images of the TZ and endocervical epithelium in human cervical tissue explants that were inoculated with or without Pil+OpaCEA or Pil+ΔOpa GC and stained for GC and E-cadherin. Scale bar, 20 μm. (TIF) [file ppat.1008136.s005.tif]
